# Involvement of tumor necrosis factor alpha in steroid-associated osteonecrosis of the femoral head: friend or foe?

**DOI:** 10.1186/s13287-018-1112-x

**Published:** 2019-01-03

**Authors:** Bin Fang, Ding Wang, Jiaqian Zheng, Qiushi Wei, Dongxiang Zhan, Yamei Liu, Xuesong Yang, Haibin Wang, Gang Li, Wei He, Liangliang Xu

**Affiliations:** 1 Key laboratory of Orthopaedics and Traumatology of Chinese Medicine, Guangzhou University of Chinese Medicine, Guangzhou, 510405 People’s Republic of China; 2 Department of Orthopaedics Surgery, The First Affiliated Hospital of Guangzhou University of Traditional Chinese Medicine, Baiyun District, Guangzhou, 510405 Guangdong People’s Republic of China; 3 Departments of Diagnostics of Traditional Chinese Medicine, Guangzhou University of Traditional Chinese Medicine, Guangzhou, People’s Republic of China; 4 Division of Histology and Embryology, Key Laboratory for Regenerative Medicine of the Ministry of Education, Medical College, Jinan University, Guangzhou, 510632 People’s Republic of China; 5 Department of Orthopaedics and Traumatology, Faculty of Medicine, Prince of Wales Hospital, The Chinese University of Hong Kong, Shatin, Hong Kong, Special Administrative Region of China; 6 Laboratory of Orthopaedics and Traumatology, Lingnan Medical Research Center, Guangzhou University of Chinese Medicine, Guangzhou, People’s Republic of China

**Keywords:** Osteonecrosis of the femoral head, Tumor necrosis factor alpha, Mesenchymal stem cells, DNA methylation

## Abstract

**Background:**

The etiology and pathology osteonecrosis of the femoral head (ONFH) are not completely clarified. As a cytokine participating in systemic inflammation, tumor necrosis factor alpha (TNFα) has been shown to be involved in the pathogenesis of ONFH. However, the role of TNFα in ONFH is not clearly clarified. In the present study, we investigated the effects of TNFα on proliferation, angiogenesis, and osteogenic differentiation of rat bone mesenchymal stem cells (rMSCs) and the underlying mechanisms.

**Methods:**

All femoral bone tissues were separated in surgeries. After extracting total RNA and protein, we evaluated TNFα content by ELISA and the relative expression levels of genes by quantitative real-time PCR and western blot. Also, immunohistochemistry staining was performed to observe the expression of Runx2 in the bone samples. Chick embryo chorioallantoic membrane (CAM) assay was performed to observe the effect of TNFα on angiogenesis. The genomic DNAs were treated by bisulfite modification, and methylation status of CpG sites in the CpG islands of human and rat Runx2 gene promoter was determined by DNA sequencing. The binding of H3K4me3 and H3K27me3 in Runx2 promoter was checked by ChIP assay. RNA-seq analysis was used to find out the genes and pathways changed by TNFα in rMSCs.

**Results:**

The results demonstrate TNFα promotes cell proliferation and angiogenesis whereas inhibits osteogenesis. Epigenetic regulations including DNA methylation and histone modifications play important roles in mediating the effect of TNFα on osteogenic differentiation. We find an increased rate of CpG methylation in rat Runx2 promoter in TNFα-treated rMSCs, as well as significantly increased occupancy of H3K27me3 in Runx2 gene promoter. The content of TNFα in necrotic tissue is much lower than that of normal tissue. And relevantly, human Runx2 promoter is demethylated in necrotic tissue using bone samples from patient with ONFH. In addition, we have observed that Wnt signaling pathway is inhibited by TNFα as multiple Wnts are markedly decreased in TNFα-treated rMSCs by RNA-seq analysis.

**Conclusion:**

Taken together, our study shows that TNFα plays complicated roles in the pathogenesis of ONFH, including proliferation, angiogenesis, and osteogenesis. Targeting TNFα should not be considered as an applicable strategy to inhibit the progression of ONFH.

**Electronic supplementary material:**

The online version of this article (10.1186/s13287-018-1112-x) contains supplementary material, which is available to authorized users.

## Introduction

Osteonecrosis of the femoral head (ONFH) is a painful disease which may lead to a total hip replacement [1], while the etiology and pathology are not completely clarified [2]. Plenty of risk factors have been recognized that contribute to ONFH, such as hypercortisonism, hyperlipidemia, autoimmune diseases, alcoholism, and clotting disturbances [3, 4]. The treatment of ONFH, particularly in collapse stages, remains challenging. Bone marrow-derived mesenchymal stem cells (BMSCs) have been emerging as a promising cell source for stem cell therapy, particularly for bone and cartilage diseases. Mesenchymal stem cells (MSCs) are characterized by their self-renewal and pluripotent differentiation capability as well as the tropism for sites of tissue damage [5]. Accumulating evidence has shown that BMSCs by intravenous infusion could migrate to the injury location to alleviate destructive inflammation and promote tissue repair, which has been confirmed in bone fracture [6], myocardial infarction [7], ischemic injuries [8], wound healings [9], and autoimmune diseases [10], making them particularly promising for therapeutic cell transplantation.

Tumor necrosis factor alpha (TNFα) has been found as a substance that mediates endotoxin-induced necrosis of tumors. It is well known as a cytokine participating in systemic inflammation and acute phase reaction. It plays a complicated role in cellular processes, including cell proliferation, differentiation, and immune reaction [11]. Produced mainly by macrophages as well as other immune cells, TNFα has been found elevated in serum and bone marrow in both animal and clinical experiments during the process of steroid-induced osteonecrosis [12]. TNFα gene polymorphisms have been suggested to be associated with the susceptibility of systemic lupus erythematosus [13], ankylosing spondylitis [14], and ONFH [15–17]. It is generally accepted that apoptosis of osteocyte in necrotic zone caused by TNFα and its receptor is one of the main reasons resulted in osteonecrosis and the destruction of the bone structure. However, the role of TNFα in ONFH is not clearly clarified.

The reductions of blood supply, osteogenesis, proliferation, and apoptosis are the main factors involved in ONFH. Wang et al. have reported that low concentrations of TNFα promote osteogenic differentiation by activating the ephrinB2-EphB4 signaling pathway [18]. However, high-dose TNFα suppressed osteogenic differentiation of BMSCs via activation of the Wnt/β-catenin signaling [19], or by depressing the activation of NF-кB signaling pathway [20]. But none of these studies have investigated the involvement of epigenetic regulation in the pathogenesis of ONFH, such as DNA methylation and histone modification. Up to now, DNA methylation and histone modifications are the most important epigenetic regulators which possess the power to control the differentiation or maintain the self-renewal of MSCs [21]. DNA methylation tends to block gene expression by incompletely known mechanisms, including the interference of the binding of transcription factors to the regulatory sites in DNA. It is known that changes in the methylation states of the CpG islands in the promoter regions or the first exon are inversely correlated with the expressions of the corresponding genes. Histone modifications can influence the interactions of transcription factors with chromatin. The analysis of histone modifications in embryonic stem cells has found many bivalent loci that are associated with both H3 lysine 27 trimethylation (H3K27me3) and H3 lysine 4 trimethylation (H3K4me3) [22–25]. The bivalent loci in MSCs are often low in DNA methylation and can be further methylated or activated, which are distinct from those in the embryonic stem cells and differentiated cells [26].

The purpose of this study is to investigate the involvement of TNFα in ONFH. Our finding clearly demonstrates that TNFα could promote cell proliferation and angiogenesis while inhibits osteogenesis of rMSCs. The lower content of TNFα in necrotic tissue as well as higher expression of Runx2, OPG, and RANKL implies that bone formation and remodeling are very active in this area. Furthermore, we find Wnt signaling pathway is significantly inhibited by TNFα through RNA-seq analysis. And epigenetic mechanisms involving both DNA methylation and histone modification contribute to the closing of the chromatin structure and inactivation of Runx2 expression caused by TNFα. These findings would help researchers better understand the role of TNFα in ONFH and develop better strategies to manage ONFH.

## Materials and methods

### Patients and ethical statement

Sixty patients who had corticosteroid usage histories and fulfilled the diagnosis of ONFH according to the guidelines of the Chinese Medical Association were recruited in the First Affiliated Hospital of Guangzhou University of Traditional Chinese Medicine. Femoral heads samples were obtained during total hip arthroplasty surgery. The study was approved by the local ethics board, and patients gave informed written consent.

### Reagents and cell culture

TNFα was purchased from PEPROTECH (USA). Rat bone marrow-derived MSCs (rMSCs) were acquired from 3-week old male Sprague-Dawley (SD) rats as described before [27]. rMSCs were maintained in α-minimal Eagle’s medium (Gibco, USA) supplemented with 10% (*v*/*v*) fetal bovine serum (Gibco, USA), 100 U/ml penicillin (Gibco, USA), and 100 μg/ml streptomycin (Gibco, USA) cultured with 5% CO_2_ at 37 °C. For osteogenic differentiation, the cells were cultured in α-MEM supplemented with 10% fetal bovine serum, 1% penicillin/streptomycin (Gibco, USA), 50 μM dexamethasone (Sigma, USA), 50 mM ascorbic acid (Sigma, USA), and 10 mM β-glycerophosphate (Sigma, USA). The medium was changed every 2 days.

### Enzyme-linked immunosorbent assay

According to the manufacturer’s instructions, the concentrations of TNFα were verified by commercial ELISA kits (R&D Systems, USA) in bone tissues. Briefly, the femoral head samples were cut into 0.5-cm-thick pieces with diamond saw (Leica). Then, normal and necrotic tissues were isolated and grinded with liquid nitrogen. The tissues were weighted and lysed with RIPA buffer (1 ml per 0.1 g tissue). At last, 10 μl lysate from each sample was used for ELISA assay. ELISA was performed strictly according to the manufacturer’s instructions. The absorbance was measured at 450 nm using a microplate reader (Wellscan MK3, Labsystems Dragon, Finland). Protein samples were quantified by a BCA Protein Assay Kit (Beyotime, Jiangsu, China). The content of TNFα was normalized by total proteins in each sample.

### Cell proliferation assay

Cells (5 × 10^3^ per well) were sub-cultured in a 96 flat-bottomed well plate. After 12 h of incubation, the medium was changed into TNFα containing media at different concentrations. Cells were incubated at 37 °C for 24 h, 48 h, and 96 h. The cell proliferation was determined using Cell-Titer 96® A-queous One Solution Cell Proliferation Assay (MTS). After incubation, cells were treated with the MTS solution (20 μl per well) for 1 h at 37 °C. Finally, formazan absorbance at 490 nm was measured in a microplate reader.

### Chick embryo chorioallantoic membrane assay

Fertilized Leghorn eggs were purchased from Avian Farm of the South China Agriculture University (Guangzhou, China). The eggs were incubated in an incubator (Yiheng Instruments, Shanghai, China) at 38 °C with 70% humidity. On incubation day 3, 2–3 ml of albumen was aspirated with a syringe needle so as to detach the developing chorioallantoic membrane (CAM) from the top part of the shell. On day 6, a window of around 1.5 cm^2^ was gently opened with a scalpel on the wide end of the egg without damaging the embryo. The window was then sealed with transparent tape to prevent dehydration and possible infections before returning to the hatching incubator. On day 8, the CAMs were implanted with 1 mm^3^ sterilized gelatin sponge containing 10 ng TNFα or PBS (control) for 96 h. On day 12, the angiogenesis was evaluated as the number of vessels converging toward the sponge which was photographed in ovo by a stereomicroscope (Olympus).

### ALP activity assay

After rMSCs were treated with or without osteogenic-induced medium (OIM) and TNFα (1 ng/ml and 10ng/ml) for 7 days, the plate was washed with PBS and the cells were lysed by lysis buffer consisting 20 mM Tri-HCl (pH 7.5), 150 mM NaCl, and 1% Triton X-100. The ALP activity was determined using alkaline phosphatase assay kit (Jiancheng Bioengineering, Nanjing, China). Absorbance at 520 nm was measured, and the protein concentration of cell lysates was measured using the BCA assay at 562 nm on a microplate spectrophotometer (Beyotime, Jiangsu, China). ALP activity was normalized according to the total protein concentration.

### ALP staining

After rMSCs were treated with or without OIM and TNFα for 7 days, ALP staining was conducted using BCIP/NBT Alkaline Phosphatase Color Development Kit (Beyotime, Jiangsu, China). Briefly, the cells were fixed with 70% ethanol and then incubated with ALP working solution (10 μl BCIP and 20 μl NBT in 3 ml ALP buffer) at 37 °C in the dark for 30 min. Then, the reaction was stopped by distilled water.

### Mineralization assay

After 14 days of osteogenic induction, rMSCs were fixed with 70% ethanol for 10 min. Then, the fixed cells were stained with 0.5% (*w*/*v*) Alizarin Red S (pH 4.1) for 15 min at room temperature and washed three times with deionized water. The calcium deposition is extracted with 10% (*w*/*v*) cetylpyridinium chloride (CPC, Sigma) and quantified by measuring the OD of the extract at 550 nm.

### RNA extraction and quantitative real-time PCR

Total RNA was extracted from cultured cells with Takara Mini BEST Universal RNA Extraction Kit (Takara), and the first-strand cDNA was synthesized using Prime Script RT Master Mix (Takara) according to the manufacturer’s instructions. Real-time PCR was performed using the CFX96 Real-Time PCR Detection System (Bio-Rad, USA). Primer sequences were shown in Additional file 1: Table S1. The relative quantification of gene expression was determined by calculating the values of 2^−ΔΔCT^, with each sample being normalized to the expression level of β-actin.

### RNA-seq and data analysis

Total RNA was obtained from the TNFα- or PBS-treated rMSCs using TRIzol Reagent (Takara, Dalian, China). The quality and integrity of total RNA samples were assessed using a 2100 Bioanalyzer or a 2200 TapeStation (Agilent Technologies) according to the manufacturer’s instructions. The preparation of whole transcriptome libraries and deep sequencing were performed by the Annoroad Gene Technology Corporation (Beijing, China). The differential genes identified by RNA-seq have been uploaded in Additional file 2: Table S2. DAVID bioinformatics tool was also used for functional annotation enrichment and clustering.

### Western blot

Whole cell lysates were prepared as described previously [28]. Protein samples were quantified by a BCA Protein Assay Kit (Beyotime, Jiangsu, China). Equal proteins were loaded onto 10% SDS-PAGE and subsequently transferred onto a polyvinylidene difluoride (PVDF) membrane (Millipore). The membrane was blocked with 5% skim milk and were incubated with anti-Runx2 (Abcam, 1:1000) or anti-β-actin (Santa Cruz, 1:1000) antibodies at 4 °C overnight, respectively. After washing with TBST for three times, the membrane was incubated with horseradish peroxidase-linked secondary antibodies. Proteins were detected with Pierce® ECL Western Blotting Substrate (Thermo Scientific) according to the manufacturer’s instruction.

### DNA isolation and bisulfite treatment

Genomic DNA was isolated from fresh bone samples. Briefly, the samples were digested with proteinase K, extracted with phenol/chloroform/isoamyl alcohol (25:24:1), precipitated with ethanol, and resuspended in TE buffer (0.1 M Tris, 1 mM Na_2_EDTA, pH 7.5). Bisulfite modification was done as described previously [29, 30]. Modified DNA was purified using Wizard DNA Clean-Up System following the manufacturer’s instructions (Promega) and eluted into 50 μL water. Modified DNA was used immediately or stored at − 20 °C.

### Bisulfite sequencing

Bisulfite-modified genomic DNA was amplified by PCR. The sequences of primers used for the bisulfite sequencing analysis were shown in Additional file 3: Table S3. PCR products were purified and cloned into pMD™19-T Vector. Colonies were selected and grown overnight in Luria-Bertani medium containing ampicillin (100 μg/ml). Plasmid DNA was isolated and sequenced using the M13 universal reverse primer (BGI, China).

### Chromatin immunoprecipitation assay

Cross-linking and chromatin immunoprecipitation (ChIP) were done as described previously [31, 32]. Briefly, all of the subsequent steps were performed at 0–4 °C, and all of the buffers contained 0.1 mM EDTA, 0.5 mM EGTA, 1 mM dithiothreitol, and protease inhibitors (BD). The rMSCs were washed with phosphate-buffered saline (pH 7.4) and lysed. After centrifugation, the pellet was resuspended in 10 ml of 10 mM Tris-HCl, pH 8.0, and 200 mM NaCl; rotated for 10 min; and centrifuged at 15,000*g* for 10 min. The chromatin pellet was resuspended in 1 ml of 50 mM Tris-HCl, pH 7.9, and 5 mM CaCl2 and digested with 500 units of micrococcal nuclease (New England Biolabs) at 37 °C for 10 min. For ChIP reactions, the samples (1 ml) were immunoprecipitated overnight with anti-H3K4me3, H3K27me3, or control rabbit IgG. ChIP-PCR analysis was done by using 3 μl of ChIP DNA and primer sets shown in Additional file 3: Table S3. For histone and Runx2 ChIPs from rMSC cells, enrichment was determined relative to a control ChIP with IgG antibody.

### Histology and immunohistochemistry

Immunohistochemical staining was performed as previously described [33, 34]. The human femur head samples were obtained during total hip arthroplasty surgery. These samples were washed in PBS, fixed in 4% paraformaldehyde, decalcified, dehydrated, and embedded in paraffin. The sections were cut at a thickness of 5 μm and were stained with H&E after deparaffination. Antigen retrieval was then performed with citrate buffer at 80 °C for 10 min for immunohistochemistry detection. Primary antibody against Runx2 protein (1:200, Abcam), anti-TNFα (1:100, Santa Cruz), and goat anti-rabbit IgG horseradish peroxidase (HRP)-conjugated secondary antibody were used for signal detection of Runx2 or TNFα. The sections were rinsed, counterstained in hematoxylin, dehydrated with graded ethanol and xylene, and mounted with p-xylene-bis-pyridinium bromide (DPX) permount (Sigma Aldrich, USA). Primary antibody was replaced with blocking solution in the negative controls. All incubation times and conditions were strictly controlled.

### Statistical analysis

Comparison of two independent groups was done using Mann-Whitney *U* test. The content of TNFα was compared by paired-samples *t* test. All data were presented as mean ± SD. All the data analysis was done using SPSS (version 16.0; SPSS Inc., Chicago, IL). *p* < 0.05 was regarded as statistically significant.

## Results

### Reduced TNFα content in necrotic tissue

**Fig. 1:**
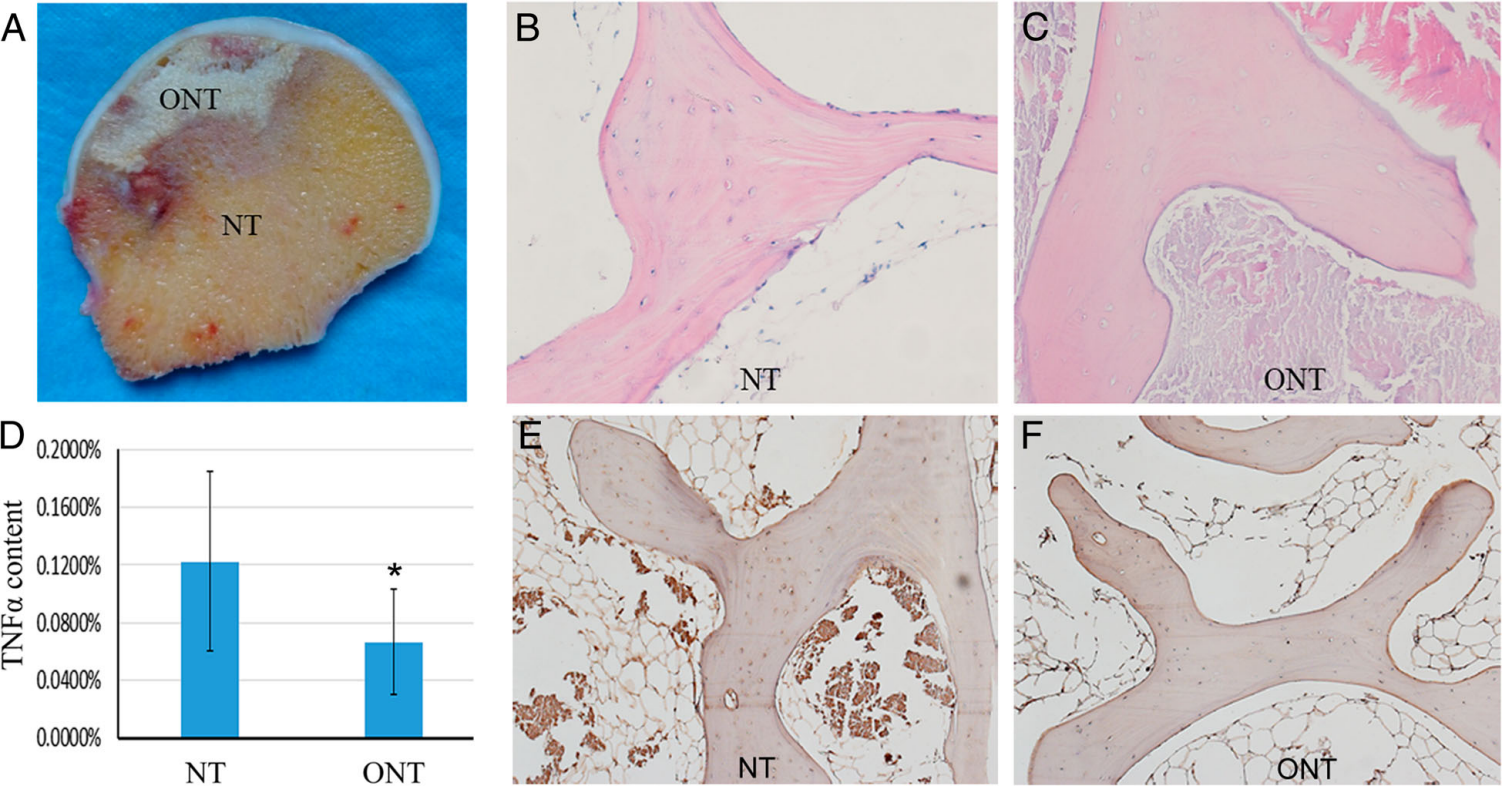
The content of TNFα in normal tissues and necrotic tissues in patients with steroid-associated ONFH. **a** The normal tissues (NT) and osteonecrotic tissues (ONT) were obtained. **b**, **c** Hematoxylin and eosin staining of the tissues, typical sign of empty lacunae was observed in ONT (magnification, × 200). **d** Relative levels of TNFα in normal tissues and necrotic tissues. The content of TNFα was measured by ELISA assay and normalized with total proteins. Data is presented as mean ± SD (*n* = 60, *p < 0.05). **e**, **f** Detection of TNFα in bone samples by immunohistochemical staining. Bone samples of NT and ONT were decalcified and sectioned. Anti-TNFα antibody was used for immunohistochemical staining (magnification, × 100)

Apoptosis of osteocytes in the necrotic zone caused by TNFα and its receptor is one of the main reasons that resulted in the osteonecrosis and the destruction of bone structure. In order to investigate the involvement of TNFα in osteonecrosis, first, we measured and compared the content of TNFα in the necrotic zone and the adjacent normal zone of human femur head samples with ONFH (Fig. 1a). HE staining demonstrated that the necrotic zone showed a typical sign of empty lacunae (Fig. 1b, c). By ELISA assay, we found the content of TNFα in the necrotic zone was significantly lower than the adjacent normal zone (Fig. 1d). The IHC staining result also showed the level of TNFα in the necrotic tissue was much higher than that in the adjacent normal tissue (Fig. 1e, f).

### TNFα promotes cell viability and angiogenesis

**Fig. 2:**
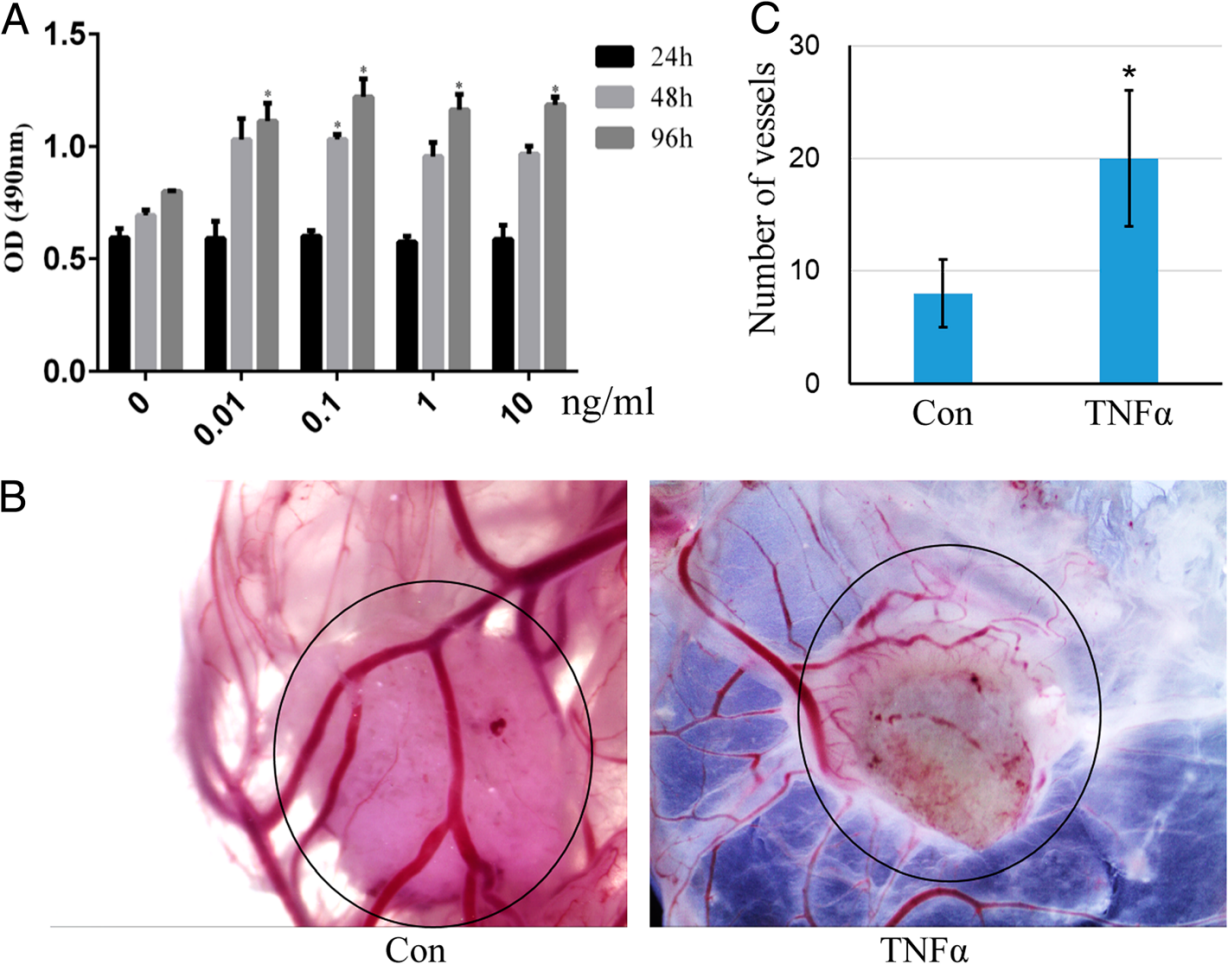
TNFα promotes rMSCs proliferation and angiogenesis. **a** The rMSCs were incubated with TNFα (0–10 ng/ml) for 1, 2, and 4 days, then the Cell Titer 96® Aqueous One Solution Cell Proliferation Assay was performed to test the cell viability. *p < 0.05 compared with 24 h. **b** PBS or TNFα (10 ng/ml) was loaded into gelatin sponge and transplanted on the chick CAM. On day 12, then the CAMs were photographed. **c** The number of vessels converging toward the sponge was counted. Data are presented as mean ± SD (*n* = 3, *p < 0.05)

Then subsequently, we checked the effect of TNFα on cell viability using the Cell Titer 96® Aqueous One Solution Cell Proliferation Assay. The result showed that TNFα could promote rMSCs proliferation when it was used at dosages between 0 and 10 ng/ml (Fig. 2a). Interrupted blood circulation in the femoral head has been considered as one of the major factors leading to osseous tissue necrosis, although the pathogenesis and etiology of ONFH have not yet been completely elucidated. So, we also evaluated the effect of TNFα on angiogenesis using the gelatin sponge-CAM culture system. One hundred microliters TNFα (10 ng/ml) was loaded into the gelatin sponge and transplanted into the CAM. On day 12, the vessels were photographed in ovo by a stereomicroscope, and the angiogenic response was evaluated as the number of vessels converging toward the sponge. The result demonstrated that TNFα enhanced angiogenesis compared with the PBS control group (Fig. 2b, c).

### TNFα inhibits osteogenesis of rMSCs

**Fig. 3:**
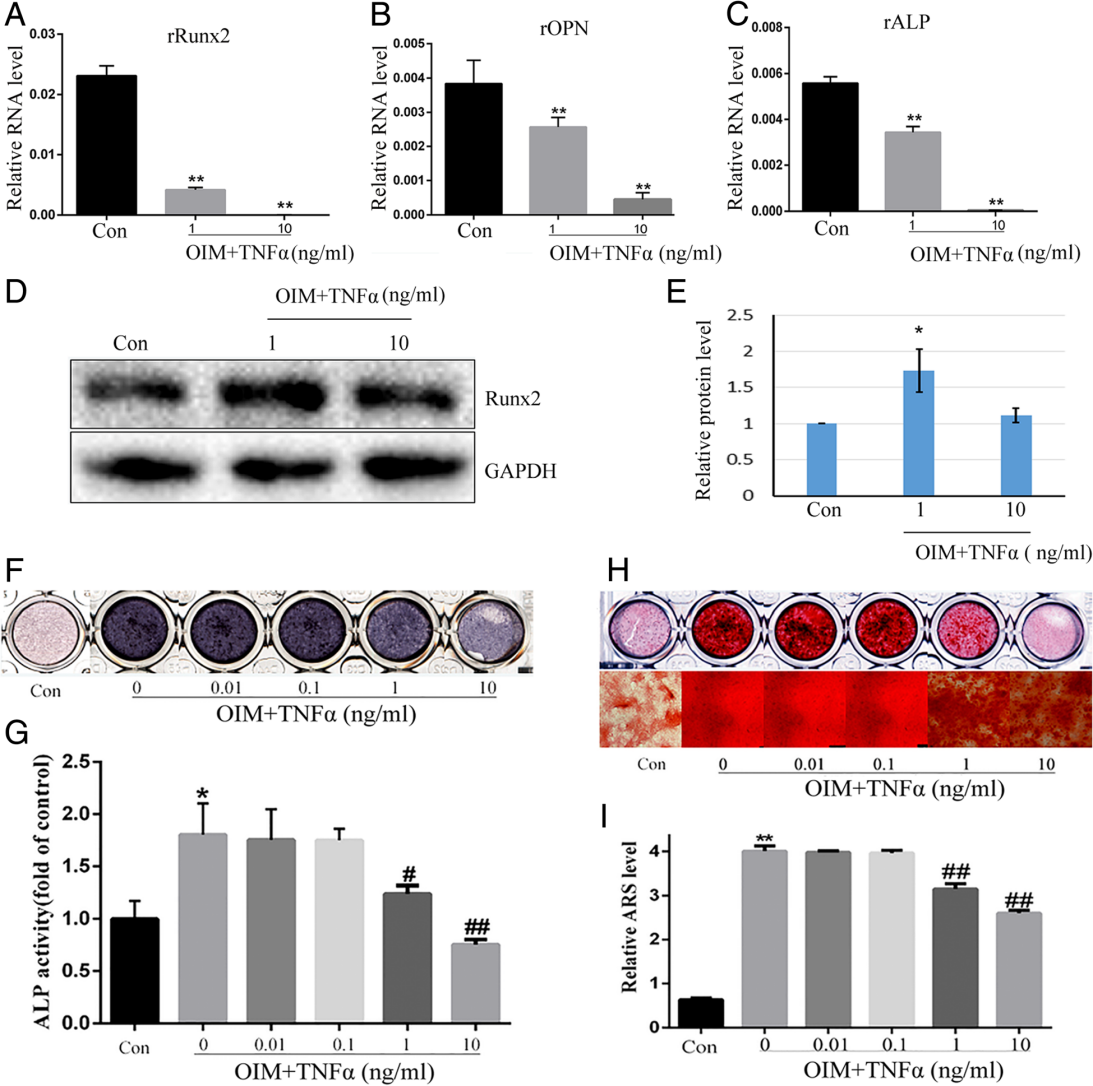
TNFα inhibits osteogenesis of rMSCs. **a–c** rMSCs were treated with TNFα (1 and 10 ng/ml) in OIM for 5 days, then gene expressions were detected by real-time PCR. β-actin was used as loading control. The experiments were repeated three times. **p < 0.01 compared with OIM. **d** Levels of Runx2 were analyzed by western blot with indicated antibodies. **e** Quantification of the band intensity using ImageJ software. Data are presented as mean ± SD (*n* = 3, *p < 0.05). **f** ALP in rMSCs was stained with BCIP/NBT kit after the cells were incubated with different concentrations of TNFα in OIM for 7 days. **g** The ALP activity of rMSCs was measured after they were incubated with different dosages of TNFα in OIM for 7 days. *p < 0.05 compared with Con (α-MEM), ^#^*p* < 0.05, ^##^*p* < 0.01 compared with OIM. **h** rMSCs were treated with TNFα (1 and 10 ng/ml) in OIM for 14 days, then the mineralized nodules were stained by Alizarin Red S. **i** The mineralization was quantified by extraction of Alizarin Red S dye with 10% CPC. **p < 0.01 compared with Con, ^##^*p* < 0.01 compared with OIM

In order to evaluate the effect of TNFα on osteogenesis, rMSCs were treated with osteogenic induction medium supplemented with different dosages of TNFα for 7 days. We detected the gene expression of Runx2, OPN, and ALP which are markers of osteoblastic differentiation. The qPCR result showed that rOPN, rALP, and rRunx2 were all significantly downregulated by TNFα at the concentration of 10 ng/ml (Fig. 3a–c). As Runx2 is the master transcription factor in osteogenesis, we also detected the protein level of Runx2 by western blot. The result revealed that TNFα significantly decreased the levels of Runx2 (Fig. 3d, e). In addition, the ALP staining result suggested that TNFα decreased ALP activity in a dose-dependent manner, and the dosage of 10 ng/ml showed to be the most effective one (Fig. 3f). And a similar result was observed by ALP activity assay (Fig. 3g).

Finally, we examined the effect of TNFα on calcium mineralization of rMSCs. Alizarin Red S staining showed that there was no calcium nodule formation in the absence of OIM at day 14; while in the presence of OIM, TNFα decreased the mineralized nodule formation (Fig. 3h). Quantification of Alizarin Red S showed that TNFα markedly decreased calcium deposition in a dose-dependent manner as compared with the control, and the maximal effect was observed at a dosage of 10 ng/ml (Fig. 3i).

### TNFα regulates rat Runx2 expression by DNA methylation and histone modification

**Fig. 4:**
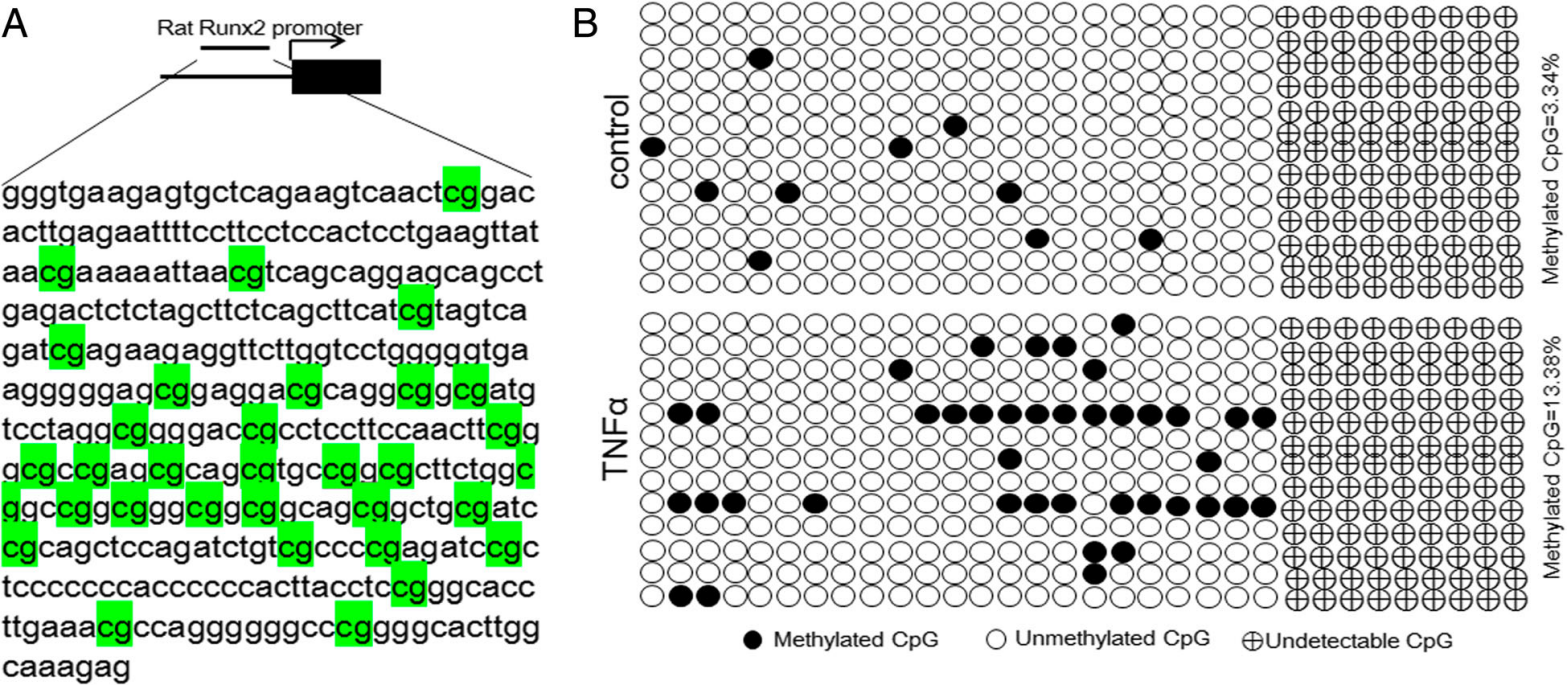
TNFα increases DNA methylation of Runx2 promoter in rMSCs. **a** Schematic figure indicates 32 CpG sites in CpG island of the rat Runx2 promoter. **b** DNA methylation status of rat Runx2 promoter in rMSCs treated with or without TNFα using sodium bisulfite sequencing. Each PCR product was subcloned and subjected to nucleotide sequencing analysis. Thirteen representatives of sequenced clones were depicted by filled (methylated) and open (unmethylated) circles for each CpG site. Nine CpG sites could not be detected

**Fig. 5:**
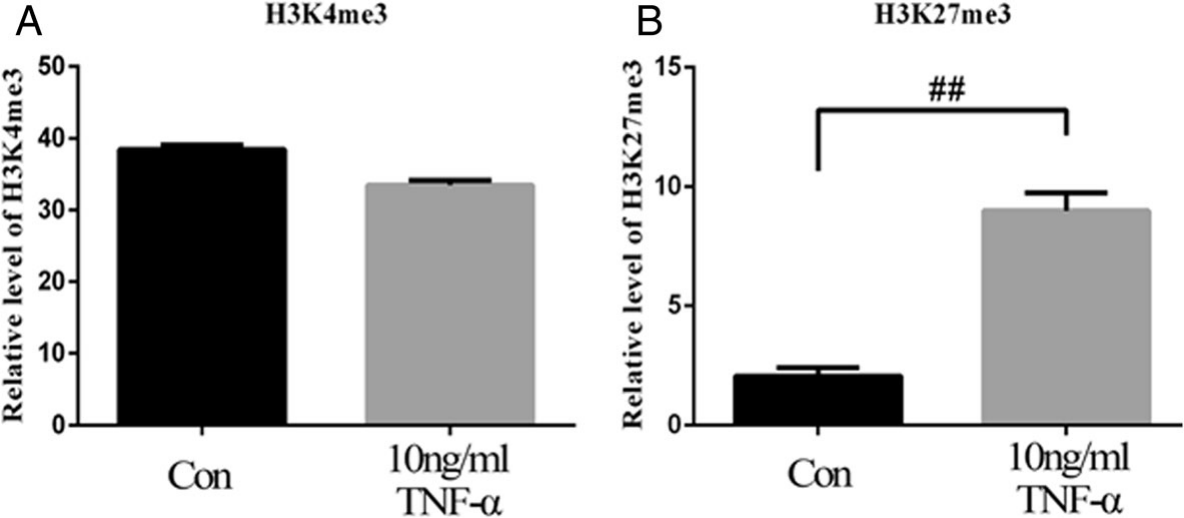
ChIP analysis of H3K4me3 and H3K27me3 in rat Runx2 promoter. **a** Decreased occupancy of H3K4me3 and **b** increased occupancy of H3K27me3 on rat Runx2 promoter in rMSCs treated with TNFα. The histone modifications of rat Runx2 promoter were analyzed by the ChIP-PCR assay. ChIP was done using anti-H3K4me3 and anti-H3K27me3 monoclonal antibodies, and PCR was performed with specific primers. Normal rabbit IgG was used as loading control. Values are expressed as mean ± SD of three independent experiments. ^##^p < 0.01

As DNA methylation and histone modification are associated with target gene expression in stem cells to regulate cell fate, we calculated the percentage of methylated CpG loci (percent CpG methylation) in the total CpG loci as well as H3K4me3 and H3K27me3 occupancy in rat Runx2 promoter. We found that rat Runx2 promoter was hypomethylated in rMSCs, and the methylation ratio of CpG sites was increased after the rMSCs were treated with TNFα at the concentration of 10 ng/ml (3.34% and 13.38% CpG methylation) (Fig. 4a, b). Then, we next performed chromatin immunoprecipitation (ChIP) assay to check the occupancy of H3K4me3 and H3K27me3 on the promoter regions of rat Runx2 with a specific monoclonal antibody. We found that occupancy of H3K27me3 (repressive histone modification) in rMSCs treated with TNFα (10 ng/ml) was significantly increased, and the activating histone modification H3K4me3 was slightly decreased (Fig. 5a, b).

### TNFα inhibits Wnt signaling in rMSCs

**Fig. 6:**
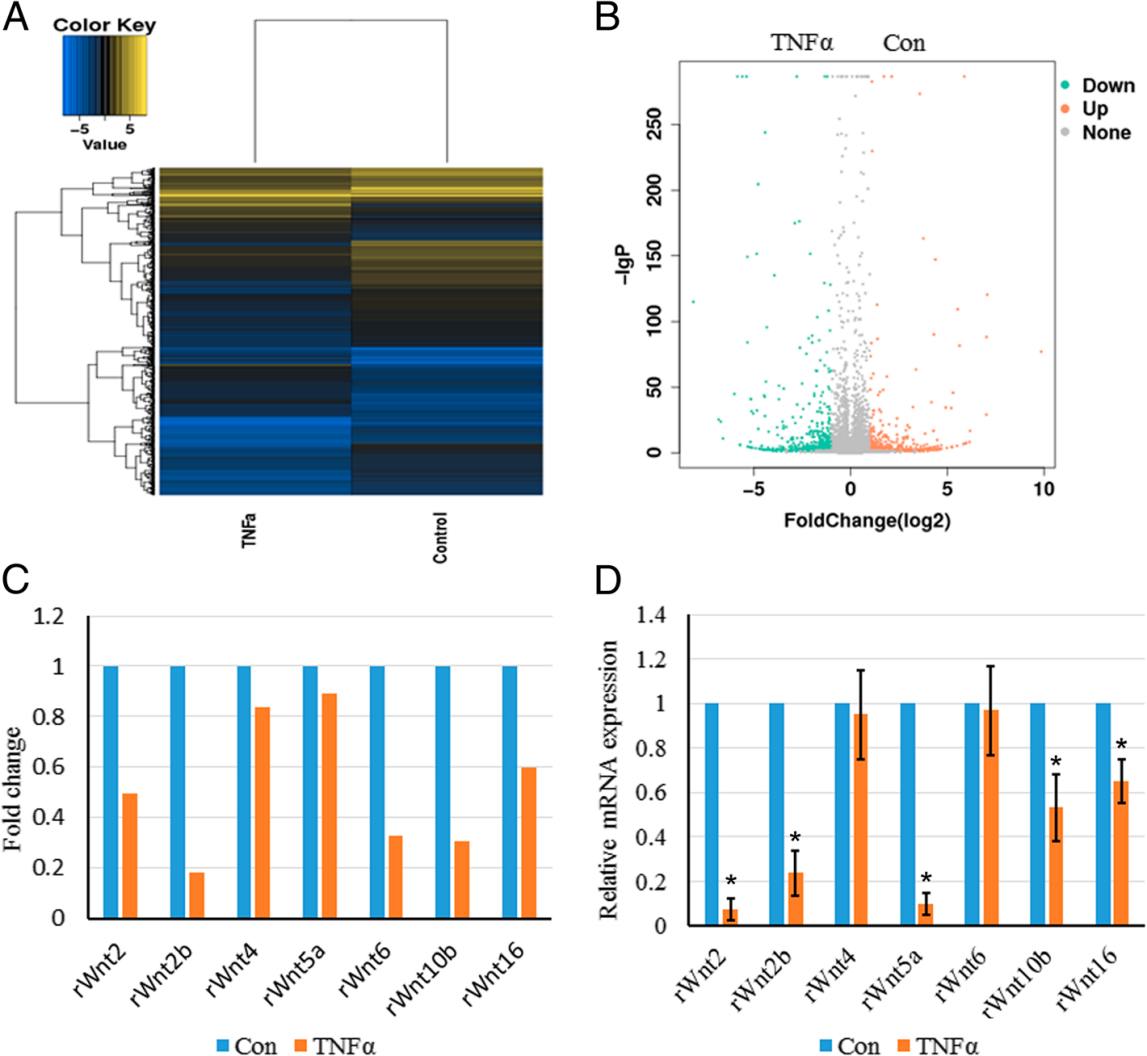
TNFα inhibits Wnt signaling in rMSCs. **a** Heatmap depicting expression levels of genes between PBS- and TNFα-treated rMSCs. **b** Volcano map of the differentially expressed genes in PBS- and TNFα-treated rMSCs. **c** Relative level of rat Wnt2/2b/4/5a/6/10b/16 in PBS- and TNFα-treated rMSCs as analyzed by RNA-seq. **d** Evaluate the expression levels of rat Wnt2/2b/4/5a/6/10b/16 in PBS- and TNFα-treated rMSCs by quantitative RT-PCR. β-actin was used as an internal control. The data are expressed as mean ± SD (*n* = 3, *p < 0.05)

To further analyze the underlying mechanism by which TNFα inhibits osteogenesis, RNA-seq was further performed to check the gene expression profiles of TNFα-treated rMSCs. The heatmap and volcano map were shown in Fig. 6a and b, respectively. One hundred ninety-three upregulated and 327 downregulated genes with log_2_ ratio above 2 or − 2 were discovered in TNFα-treated rMSCs vs control rMSCs. The Kyoto Encyclopedia of Genes and Genomes (KEGG) analysis revealed that several signaling pathways were enriched as shown in Additional file 4: Figure S1, among which we found that Wnt signaling was the most attractive for further study as it was indispensable for osteogenesis and bone development. The RNA-seq data showed that Wnt2/2b/4/5a/10b/16 were downregulated by TNFα (Fig. 6c). And this finding was further confirmed by quantitative real-time PCR (Fig. 6d).

### Higher level of human Runx2 expression in the necrotic area is associated with demethylation

**Fig. 7:**
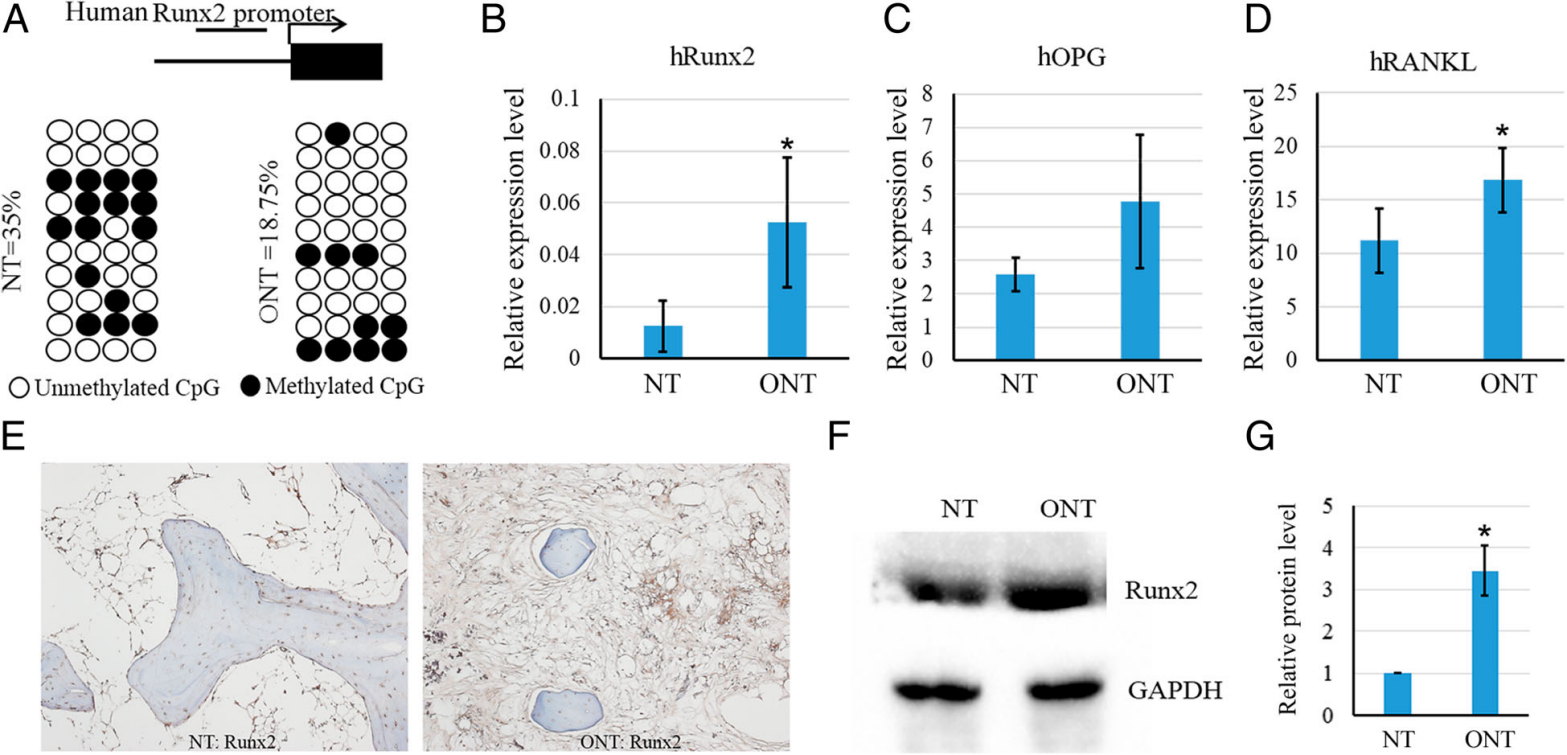
DNA methylation influences human Runx2 expression in patients with steroid-associated ONFH. **a** DNA methylation status of human Runx2 promoter in normal tissues and necrotic tissues in patients with steroid-associated ONFH using sodium bisulfite sequencing. Each PCR product was subcloned and subjected to nucleotide sequencing analysis. Ten representatives of sequenced clones were depicted by filled (methylated) and open (unmethylated) circles for each CpG site. **b**–**d** The expression levels of human Runx2, OPG and RANKL were detected by quantitative RT-PCR. β-actin was used as loading control. The experiments were repeated three times (*n* = 16). **e** Detection of human Runx2 expression in human bone samples by immunohistochemical staining. Bone samples of NT and ONT were decalcified and sectioned. Anti-Runx2 antibody was used for immunohistochemical staining. **f** The protein expression levels of human Runx2 in necrotic tissues and normal tissues were detected by western blot with indicated antibodies. NT, normal tissue; ONT, osteonecrotic tissue. **g** Quantification of the band intensity using ImageJ software. Data are presented as mean ± SD (*n* = 3, *p < 0.05)

In order to confirm our finding that Runx2 is epigenetically regulated by TNFα in patients with ONFH, the human Runx2 promoter was analyzed by bisulfite sequencing in normal tissue and necrotic tissue obtained from patients with ONFH. One major characteristic of human Runx2 is that it does not have a CpG island in its promoter, which is different with rat Runx2 promoter. Four CpG sites were chosen by MethPrimer in human Runx2 promoter for bisulfite sequencing analysis. The result demonstrated that CpG sites in human Runx2 promoter were significantly demethylated in necrotic tissue, compared with the methylation rate in normal tissue (35%) (Fig. 7a). As expected, the mRNA expression level of hRunx2 in the necrotic area was significantly higher than that of normal tissue (Fig. 7b). Also, the relative expression levels of hOPG and hRANKL were both increased in necrotic tissue (Fig. 7c, d). And apart from that, Runx2 expression was detected by immunohistochemical staining in human bone samples of normal tissue (NT) and osteonecrotic tissue (ONT) (Fig. 7e), and western blot showing the protein level of Runx2 in necrotic tissue was higher than that in normal tissue (Fig. 7f, g).

## Discussion

In the present study, we found that the content of TNFα in necrotic tissue was much lower than that in normal tissue. Our results indicated that TNFα, especially at the concentration of 10 ng/ml, had a negative effect on the mineralization of rMSCs and Wnt signaling pathway. However, on the other hand, the proliferation of rMSCs and angiogenesis were promoted by TNFα. This is a very interesting finding as TNFα exists as both friend and foe in the pathogenesis of ONFH, especially at the recovery stage. Our finding demonstrates that targeting TNFα should not be considered as an applicable strategy to inhibit the progression of ONFH.

TNFα has been previously reported to be elevated in serum and bone marrow in both animal and clinical experiments during the process of steroid-induced osteonecrosis [12]. However, in our study, we observed lower TNFα content in the necrotic area compared with the normal tissue of the femur head. The possible reason could be TNFα is only increased at the initial stage of ONFH. The increase of TNFα could promote cell proliferation and angiogenesis while inhibiting osteoblastic differentiation of rMSCs and result in decreased mineralization. Although the pathogenesis and etiology of ONFH have not yet been completely elucidated, interrupted blood circulation in the femoral head has been accepted as one of the main causes leading to osseous tissue necrosis. Our result from CAM assay indicated that the existence of TNFα could benefit angiogenesis which may help prevent the progress of ONFH. This finding was consistent with the previous report that TNFα and LPS could lead to the upregulation of VEGF and SIRT1, and subsequent upregulation of MMP-2 and MMP-9 production, and promote angiogenesis via pathways involving PI3K, p38, ERK, JNK, and NF-κB [35].

Several earlier studies confirmed that TNFα had a negative effect on osteoblast differentiation [36, 37]. However, the influence of TNFα on osteogenic differentiation of MSCs remains controversial. Recent researches have also found that TNFα enhances osteogenic differentiation of adipose-derived stem cells via the activation of NF-κB and increasing of PDZ-binding motif expression [38]. Besides, low concentration TNFα had been confirmed to increase BMP-2 expression in MSCs [39]. Studies showed that the osteogenic differentiation of rMSCs is inhibited by TNF-a, which is demonstrated by decreased levels of ALP, Runx2, and OPN after treated with low concentration TNFα for 14 days during osteogenic differentiation [40, 41]. Our data also found that high-dose TNFα (10 ng/ml) displayed a negative effect on osteogenic differentiation of rMSCs. ALP has been reported to enhance mineralization by catalyzing the hydrolysis of organic phosphate esters, thereby providing inorganic phosphates for mineralization [42]. ALP staining results indicated that the ALP-positive cells ratio was significantly decreased upon treatment with high concentration TNFα. RT-PCR analysis also showed that TNFα treatment suppressed the expression of Runx2, ALP, and OPN. Besides, studies reported that TNFα induced rMSCs proliferation [43]. TNFα has been reported to promote cell death in osteoblast culture [44]. So, we suspected that the different effect of calcium accumulation in the presence of low and high concentration TNFα may have been due to the proliferation.

Although many studies have been conducted to evaluate the effect of TNFα on osteogenesis of MSCs, few studies have focused on the roles of epigenetic regulation in controlling the fate of MSCs. As we know, DNA methylation is one of the most frequent epigenetic mechanisms controlling gene expression in mammalian cells [45]. In this study, using bisulfite sequencing analysis, we calculated the percentage of methylated CpG loci (percent CpG methylation) in the total CpG loci in the Runx2 promoter. We found the methylated CpG sites in Runx2 promoter was increased after rMSCs were treated with TNFα at the concentration of 10 ng/ml. Furthermore, we also checked the methylation status of CpG sites of Runx2 promoter in human bone samples obtained from patients with ONFH. The result showed that Runx2 promoter was demethylated in necrotic tissue compared with the normal tissue, which is consistent with our finding that TNFα content in necrotic tissue is much lower. Taken together, these data suggest that DNA methylation could be involved, at least partially, in the regulation of osteogenic gene expression of rMSCs.

Histone modification is a principal epigenetic machinery linked to the establishment and maintenance of transcriptional states of genes [46, 47]. Histone-modifying enzymes are involved in the addition or removal of histone modifications and reciprocally collaborate to compile the complex “histone code” to fine-tune epigenetic context at a specific regulatory region, modulating the gene expression [48]. In ES cells, genes that are involved in early lineage commitment maintain both repressive (H3K27me3) and activating (H3K4me3) histone modifications [22]. These bivalent genes are considered to be poised for rapid activation in response to appropriate differentiation signals [49]. To further elucidate the role of histone modification in specific gene regulation, we examined histone H3 methylation in K4 and K27 on the promoter regions of Runx2 in rMSCs treated with TNFα or not. We performed chromatin immunoprecipitation (ChIP) assay to check the occupancy of H3K4me3 and H3K27me3 on the promoter regions of Runx2 with a specific monoclonal antibody. Compared to control rMSCs, TNFα-treated rMSCs showed significantly increased histone H3 methylation levels in K27, but not K4, implying the suppression of Runx2 gene transcription. On top of that, we have observed that multiple Wnts, such as Wnt2, Wnt2b, Wnt5a, Wnt10b, and Wnt16, are markedly decreased in TNFα-treated rMSCs by RNA-seq analysis, implying that Wnt signaling pathway is inhibited by TNFα. The Wnt signaling pathway is also involved in epigenetic regulations [50], so the inhibited Wnt signaling may also regulate the DNA methylation and histone modifications in Runx2 promoter.

## Conclusion

In summary, the present study has shown that epigenetic mechanisms involving both DNA methylation and histone modification at Runx2 promoter endowed rMSCs with epigenetic plasticity when cells were treated with TNFα. Our study provided important clues regarding the effects of TNFα on proliferation, angiogenesis, and osteogenesis, and mechanically showed epigenetic regulations involved in osteogenesis of rMSCs. These finding may assist us to better understand the role of TNFα in the pathogenesis of ONFH and develop and recognize reagents that are able to efficiently promote osteogenic differentiation and angiogenesis to accelerate recovery from ONFH.

## Additional files

Additional file 1: **Table S1.** Primers for qRT-PCR. (DOCX 15 kb)

Additional file 2: **Table S2.** Differentially expressed genes in TNFα-treated rMSCs. (DOCX 42 kb)

Additional file 3: **Table S3.** Specific primers for bisulfite sequencing PCR and ChIP-PCR. (DOCX 15 kb)

Additional file 4: **Figure S1.** The KEGG (Kyoto Encyclopedia of Genes and Genomes) analysis of enriched signaling pathways in TNFα-treated rMSCs. (TIF 562 kb)

## Abbreviations

CAM: Chorioallantoic membrane
ChIP: Chromatin immunoprecipitation
CPC: Cetylpyridinium chloride
H3K27me3: H3 lysine 27 trimethylation
H3K4me3: H3 lysine 4 trimethylation
OIM: Osteogenic induced medium
ONFH: Osteonecrosis of the femoral head
PVDF: Polyvinylidene difluoride
rMSCs: Rat bone mesenchymal stem cells
TNFα: Tumor necrosis factor alpha
