# Supplementary material for: Involvement of tumor necrosis factor alpha in steroid-associated osteonecrosis of the femoral head: friend or foe?
Source: Stem Cell Res Ther. 2019 Jan 3;10:5. doi: 10.1186/s13287-018-1112-x (PMC6318982; doi:10.1186/s13287-018-1112-x)
Supplement: Supplementary file 1 — Table S1. Primers for qRT-PCR. (DOCX 15 kb) [file 13287_2018_1112_MOESM1_ESM.docx]

**Table S1. Primers for qRT-PCR**

| Primer Name | Sequence（5'-3'） |
| --- | --- |
| rRunx2-F | CCGATGGGACCGTGGTT |
| rRunx2-R | CAGCAGAGGCATTTCGTAGCT |
| rOPN-F | TCCAAGGAGTATAAGCAGCGGGCCA |
| rOPN-R | CTCTTAGGGTCTAGGACTAGCTTCT |
| rALP-F | TCCGTGGGTCGGATTCCT |
| rALP-R | GCCGGCCCAAGAGAGAA |
| rβ-actin-F | CGTAAAGACCTCTATGCCAACA |
| rβ-actin-R | CGGACTCATCGTACTCCTGCT |
| rWnt2-F | CATCGCTGGAACTGCAACAC |
| rWnt2-R | ATCTACAAATGCACGGGCGA |
| rWnt2b-F | GGTCCATGCTATCACTCGGG |
| rWnt2b-R | TCCAGCTTCAGAAATCGCCG |
| rWnt5a-F | GGGCACATTTCCACGCTATAC |
| rWnt5a-R | GAAGACATGGCACCTCCAGC |
| rWnt6-F | GGACGTGGAGATATCCGTGC |
| rWnt6-R | AGAGCACAGGAACCCGAAAG |
| rWnt8b-F | GCCCTAGAGACAGGACAGGA |
| rWnt8b-R | TCGCGTGTAGAGATGGAACG |
| rWnt10b-F | GTTCAGTCGGGCTCTAAGCA |
| rWnt10b-R | AGCATGGAGAAGGAGAACGC |
| rWnt16-F | CTCCCAACTACTGCGTGGAG |
| rWnt16-R | TGGGTAATCCAACTGTGAGCC |
