# Supplementary material for: Involvement of tumor necrosis factor alpha in steroid-associated osteonecrosis of the femoral head: friend or foe?
Source: Stem Cell Res Ther. 2019 Jan 3;10:5. doi: 10.1186/s13287-018-1112-x (PMC6318982; doi:10.1186/s13287-018-1112-x)
Supplement: Supplementary file 2 — Table S2. Differentially expressed genes in TNFα-treated rMSCs. (DOCX 42 kb) [file 13287_2018_1112_MOESM2_ESM.docx]

**Table S2**. **Differentially expressed genes in TNFα-treated rMSCs.**

| **Gene** | **TNFa_count** | **T_normalize** | **Control_count** | | **C_normalize** | **FoldChange** | **Log2FoldChange** |
| --- | --- | --- | --- | --- | --- | --- | --- |
| LOC108348144 | 469 | 18.40164706 | | 0 | 0.019829192 | 928.0079056 | 9.857993285 |
| Nhp2 | 540 | 21.18739747 | | 4 | 0.158633537 | 133.5619054 | 7.06136477 |
| LOC103690039 | 396 | 15.53742481 | | 3 | 0.118975153 | 130.5938631 | 7.028943292 |
| Ino80b | 131 | 5.139905682 | | 1 | 0.039658384 | 129.6045156 | 7.017972174 |
| Rsl1d1l1 | 73 | 2.86422225 | | 1 | 0.039658384 | 72.22236366 | 6.174373732 |
| LOC103690118 | 36 | 1.412493165 | | 0 | 0.019829192 | 71.23301621 | 6.154474174 |
| LOC103691744 | 32 | 1.25554948 | | 0 | 0.019829192 | 63.31823663 | 5.984549173 |
| Mif | 1615 | 63.3660128 | | 27 | 1.070776375 | 59.17763435 | 5.88698012 |
| LOC100910501 | 29 | 1.137841716 | | 0 | 0.019829192 | 57.38215195 | 5.842530168 |
| LOC100911186 | 352 | 13.81104428 | | 7 | 0.27760869 | 49.75004307 | 5.636625869 |
| Pdcd5 | 470 | 18.44088298 | | 10 | 0.396583843 | 46.49933003 | 5.539138025 |
| LOC103690014 | 199 | 7.807948327 | | 5 | 0.198291921 | 39.37602841 | 5.299245699 |
| Wbp1 | 149 | 5.846152265 | | 4 | 0.158633537 | 36.85319242 | 5.203717693 |
| LOC100911660 | 16 | 0.62777474 | | 0 | 0.019829192 | 31.65911832 | 4.984549173 |
| Lemd2 | 153 | 6.00309595 | | 5 | 0.198291921 | 30.27403189 | 4.920008921 |
| LOC108350142 | 26 | 1.020133952 | | 1 | 0.039658384 | 25.72303363 | 4.684988891 |
| LOC367277 | 12 | 0.470831055 | | 0 | 0.019829192 | 23.74433874 | 4.569511674 |
| Nanos3 | 12 | 0.470831055 | | 0 | 0.019829192 | 23.74433874 | 4.569511674 |
| Triap1 | 12 | 0.470831055 | | 0 | 0.019829192 | 23.74433874 | 4.569511674 |
| Zfp420 | 45 | 1.765616456 | | 2 | 0.079316769 | 22.26031757 | 4.476402269 |
| LOC102550193 | 11 | 0.431595134 | | 0 | 0.019829192 | 21.76564384 | 4.443980792 |
| Pasd1 | 11 | 0.431595134 | | 0 | 0.019829192 | 21.76564384 | 4.443980792 |
| LOC100911685 | 677 | 26.56271868 | | 32 | 1.269068296 | 20.93088193 | 4.387561196 |
| LOC102556337 | 420 | 16.47908692 | | 21 | 0.83282607 | 19.78694895 | 4.306477268 |
| Abhd10 | 79 | 3.099637778 | | 4 | 0.158633537 | 19.53961209 | 4.288329921 |
| LOC100910418 | 183 | 7.180173587 | | 10 | 0.396583843 | 18.10505829 | 4.178320916 |
| Hcrtr2 | 9 | 0.353123291 | | 0 | 0.019829192 | 17.80825405 | 4.154474174 |
| LOC102552549 | 9 | 0.353123291 | | 0 | 0.019829192 | 17.80825405 | 4.154474174 |
| Atp12a | 8 | 0.31388737 | | 0 | 0.019829192 | 15.82955916 | 3.984549173 |
| Col10a1 | 8 | 0.31388737 | | 0 | 0.019829192 | 15.82955916 | 3.984549173 |
| Natd1 | 8 | 0.31388737 | | 0 | 0.019829192 | 15.82955916 | 3.984549173 |
| Card10 | 15 | 0.588538819 | | 1 | 0.039658384 | 14.84021171 | 3.891439769 |
| Rin1 | 73 | 2.86422225 | | 5 | 0.198291921 | 14.44447273 | 3.852445637 |
| LOC103690050 | 28 | 1.098605795 | | 2 | 0.079316769 | 13.85086426 | 3.791904095 |
| Capn3 | 14 | 0.549302897 | | 1 | 0.039658384 | 13.85086426 | 3.791904095 |
| LOC102547046 | 7 | 0.274651449 | | 0 | 0.019829192 | 13.85086426 | 3.791904095 |
| LOC103694906 | 7 | 0.274651449 | | 0 | 0.019829192 | 13.85086426 | 3.791904095 |
| LOC100909750 | 822 | 32.25192726 | | 60 | 2.379503056 | 13.55406003 | 3.760653161 |
| Scn1a | 25 | 0.980898031 | | 2 | 0.079316769 | 12.36684309 | 3.628405363 |
| LOC100911261 | 1425 | 55.91118777 | | 118 | 4.679689343 | 11.94762807 | 3.578652327 |
| Kcna2 | 12 | 0.470831055 | | 1 | 0.039658384 | 11.87216937 | 3.569511674 |
| Dnajc5b | 6 | 0.235415527 | | 0 | 0.019829192 | 11.87216937 | 3.569511674 |
| Kcnj8 | 6 | 0.235415527 | | 0 | 0.019829192 | 11.87216937 | 3.569511674 |
| LOC108350444 | 6 | 0.235415527 | | 0 | 0.019829192 | 11.87216937 | 3.569511674 |
| LOC684208 | 6 | 0.235415527 | | 0 | 0.019829192 | 11.87216937 | 3.569511674 |
| Pax3 | 6 | 0.235415527 | | 0 | 0.019829192 | 11.87216937 | 3.569511674 |
| Trnai-uau | 6 | 0.235415527 | | 0 | 0.019829192 | 11.87216937 | 3.569511674 |
| LOC100912599 | 33 | 1.294785401 | | 3 | 0.118975153 | 10.88282192 | 3.443980792 |
| Gdf10 | 11 | 0.431595134 | | 1 | 0.039658384 | 10.88282192 | 3.443980792 |
| LOC100911865 | 11 | 0.431595134 | | 1 | 0.039658384 | 10.88282192 | 3.443980792 |
| Fam134b | 348 | 13.65410059 | | 33 | 1.308726681 | 10.43311854 | 3.383098549 |
| Nxf2 | 21 | 0.823954346 | | 2 | 0.079316769 | 10.3881482 | 3.376866596 |
| Grin1 | 10 | 0.392359212 | | 1 | 0.039658384 | 9.893474474 | 3.306477268 |
| Adamts16 | 5 | 0.196179606 | | 0 | 0.019829192 | 9.893474474 | 3.306477268 |
| Aldh1a1 | 5 | 0.196179606 | | 0 | 0.019829192 | 9.893474474 | 3.306477268 |
| Jakmip2 | 5 | 0.196179606 | | 0 | 0.019829192 | 9.893474474 | 3.306477268 |
| LOC103690095 | 5 | 0.196179606 | | 0 | 0.019829192 | 9.893474474 | 3.306477268 |
| LOC103690847 | 5 | 0.196179606 | | 0 | 0.019829192 | 9.893474474 | 3.306477268 |
| LOC103693173 | 5 | 0.196179606 | | 0 | 0.019829192 | 9.893474474 | 3.306477268 |
| LOC108352427 | 5 | 0.196179606 | | 0 | 0.019829192 | 9.893474474 | 3.306477268 |
| LOC365102 | 5 | 0.196179606 | | 0 | 0.019829192 | 9.893474474 | 3.306477268 |
| Mir212 | 5 | 0.196179606 | | 0 | 0.019829192 | 9.893474474 | 3.306477268 |
| RGD1565590 | 5 | 0.196179606 | | 0 | 0.019829192 | 9.893474474 | 3.306477268 |
| RGD1565989 | 5 | 0.196179606 | | 0 | 0.019829192 | 9.893474474 | 3.306477268 |
| Slc35g2 | 5 | 0.196179606 | | 0 | 0.019829192 | 9.893474474 | 3.306477268 |
| Spata32 | 5 | 0.196179606 | | 0 | 0.019829192 | 9.893474474 | 3.306477268 |
| Syndig1l | 5 | 0.196179606 | | 0 | 0.019829192 | 9.893474474 | 3.306477268 |
| Tbx6 | 5 | 0.196179606 | | 0 | 0.019829192 | 9.893474474 | 3.306477268 |
| Trim38 | 5 | 0.196179606 | | 0 | 0.019829192 | 9.893474474 | 3.306477268 |
| Trnad-guc | 5 | 0.196179606 | | 0 | 0.019829192 | 9.893474474 | 3.306477268 |
| LOC685933 | 39 | 1.530200928 | | 4 | 0.158633537 | 9.646137612 | 3.269951392 |
| LOC103690064 | 57 | 2.236447511 | | 6 | 0.237950306 | 9.39880075 | 3.232476686 |
| Pak2 | 199 | 7.807948327 | | 21 | 0.83282607 | 9.375244859 | 3.228856371 |
| Mrgbp | 18 | 0.706246582 | | 2 | 0.079316769 | 8.904127026 | 3.154474174 |
| Chrm2 | 9 | 0.353123291 | | 1 | 0.039658384 | 8.904127026 | 3.154474174 |
| Hoxd11 | 9 | 0.353123291 | | 1 | 0.039658384 | 8.904127026 | 3.154474174 |
| LOC108351437 | 9 | 0.353123291 | | 1 | 0.039658384 | 8.904127026 | 3.154474174 |
| Hells | 116 | 4.551366864 | | 14 | 0.55521738 | 8.197450278 | 3.035175246 |
| Adcyap1r1 | 8 | 0.31388737 | | 1 | 0.039658384 | 7.914779579 | 2.984549173 |
| Fbxo16 | 8 | 0.31388737 | | 1 | 0.039658384 | 7.914779579 | 2.984549173 |
| Gja6 | 8 | 0.31388737 | | 1 | 0.039658384 | 7.914779579 | 2.984549173 |
| Mir101a | 8 | 0.31388737 | | 1 | 0.039658384 | 7.914779579 | 2.984549173 |
| Pcdh11x | 8 | 0.31388737 | | 1 | 0.039658384 | 7.914779579 | 2.984549173 |
| Tnnc2 | 8 | 0.31388737 | | 1 | 0.039658384 | 7.914779579 | 2.984549173 |
| LOC103690054 | 132 | 5.179141604 | | 17 | 0.674192532 | 7.681991944 | 2.941480451 |
| Tmem196 | 69 | 2.707278566 | | 9 | 0.356925458 | 7.584997097 | 2.923148628 |
| LOC102553715 | 110 | 4.315951336 | | 15 | 0.594875764 | 7.255214614 | 2.859018291 |
| LOC100909794 | 78 | 3.060401857 | | 11 | 0.436242227 | 7.015372809 | 2.810519773 |
| Rgs5 | 14 | 0.549302897 | | 2 | 0.079316769 | 6.925432132 | 2.791904095 |
| Crb1 | 7 | 0.274651449 | | 1 | 0.039658384 | 6.925432132 | 2.791904095 |
| Cyp2d2 | 7 | 0.274651449 | | 1 | 0.039658384 | 6.925432132 | 2.791904095 |
| Gpc5 | 7 | 0.274651449 | | 1 | 0.039658384 | 6.925432132 | 2.791904095 |
| LOC102554525 | 7 | 0.274651449 | | 1 | 0.039658384 | 6.925432132 | 2.791904095 |
| LOC108352984 | 7 | 0.274651449 | | 1 | 0.039658384 | 6.925432132 | 2.791904095 |
| LOC690686 | 7 | 0.274651449 | | 1 | 0.039658384 | 6.925432132 | 2.791904095 |
| Sec61g-ps1 | 7 | 0.274651449 | | 1 | 0.039658384 | 6.925432132 | 2.791904095 |
| Sh2d4b | 7 | 0.274651449 | | 1 | 0.039658384 | 6.925432132 | 2.791904095 |
| Mcf2 | 68 | 2.668042644 | | 10 | 0.396583843 | 6.727562642 | 2.750083919 |
| LOC500959 | 20 | 0.784718425 | | 3 | 0.118975153 | 6.595649649 | 2.721514767 |
| LOC102555260 | 13 | 0.510066976 | | 2 | 0.079316769 | 6.430758408 | 2.684988891 |
| Six3 | 13 | 0.510066976 | | 2 | 0.079316769 | 6.430758408 | 2.684988891 |
| Dcdc2 | 32 | 1.25554948 | | 5 | 0.198291921 | 6.331823663 | 2.662621078 |
| Clcn2 | 44 | 1.726380535 | | 7 | 0.27760869 | 6.218755384 | 2.636625869 |
| Gzmb | 36 | 1.412493165 | | 6 | 0.237950306 | 5.936084684 | 2.569511674 |
| LOC100910528 | 12 | 0.470831055 | | 2 | 0.079316769 | 5.936084684 | 2.569511674 |
| LOC103690005 | 12 | 0.470831055 | | 2 | 0.079316769 | 5.936084684 | 2.569511674 |
| Abca8a | 6 | 0.235415527 | | 1 | 0.039658384 | 5.936084684 | 2.569511674 |
| Acot1 | 6 | 0.235415527 | | 1 | 0.039658384 | 5.936084684 | 2.569511674 |
| Aipl1 | 6 | 0.235415527 | | 1 | 0.039658384 | 5.936084684 | 2.569511674 |
| Arhgef15 | 6 | 0.235415527 | | 1 | 0.039658384 | 5.936084684 | 2.569511674 |
| Frrs1l | 6 | 0.235415527 | | 1 | 0.039658384 | 5.936084684 | 2.569511674 |
| LOC100362479 | 6 | 0.235415527 | | 1 | 0.039658384 | 5.936084684 | 2.569511674 |
| LOC100909828 | 6 | 0.235415527 | | 1 | 0.039658384 | 5.936084684 | 2.569511674 |
| LOC100911572 | 6 | 0.235415527 | | 1 | 0.039658384 | 5.936084684 | 2.569511674 |
| LOC102547753 | 6 | 0.235415527 | | 1 | 0.039658384 | 5.936084684 | 2.569511674 |
| LOC102549615 | 6 | 0.235415527 | | 1 | 0.039658384 | 5.936084684 | 2.569511674 |
| LOC103693243 | 6 | 0.235415527 | | 1 | 0.039658384 | 5.936084684 | 2.569511674 |
| LOC103693323 | 6 | 0.235415527 | | 1 | 0.039658384 | 5.936084684 | 2.569511674 |
| LOC103693502 | 6 | 0.235415527 | | 1 | 0.039658384 | 5.936084684 | 2.569511674 |
| LOC288481 | 6 | 0.235415527 | | 1 | 0.039658384 | 5.936084684 | 2.569511674 |
| LOC681241 | 6 | 0.235415527 | | 1 | 0.039658384 | 5.936084684 | 2.569511674 |
| LOC684557 | 6 | 0.235415527 | | 1 | 0.039658384 | 5.936084684 | 2.569511674 |
| LOC685138 | 6 | 0.235415527 | | 1 | 0.039658384 | 5.936084684 | 2.569511674 |
| Mir218-2 | 6 | 0.235415527 | | 1 | 0.039658384 | 5.936084684 | 2.569511674 |
| Plcd4 | 6 | 0.235415527 | | 1 | 0.039658384 | 5.936084684 | 2.569511674 |
| R3hdml | 6 | 0.235415527 | | 1 | 0.039658384 | 5.936084684 | 2.569511674 |
| RGD1565661 | 6 | 0.235415527 | | 1 | 0.039658384 | 5.936084684 | 2.569511674 |
| Sv2b | 6 | 0.235415527 | | 1 | 0.039658384 | 5.936084684 | 2.569511674 |
| Txndc2 | 6 | 0.235415527 | | 1 | 0.039658384 | 5.936084684 | 2.569511674 |
| Upk3b | 6 | 0.235415527 | | 1 | 0.039658384 | 5.936084684 | 2.569511674 |
| Dmp1 | 29 | 1.137841716 | | 5 | 0.198291921 | 5.738215195 | 2.520602073 |
| Nmu | 28 | 1.098605795 | | 5 | 0.198291921 | 5.540345705 | 2.469976 |
| Cfap157 | 11 | 0.431595134 | | 2 | 0.079316769 | 5.441410961 | 2.443980792 |
| LOC102550577 | 11 | 0.431595134 | | 2 | 0.079316769 | 5.441410961 | 2.443980792 |
| LOC100910790 | 81 | 3.17810962 | | 15 | 0.594875764 | 5.342476216 | 2.41750858 |
| LOC100362814 | 107 | 4.198243573 | | 20 | 0.793167685 | 5.293008844 | 2.404088064 |
| LOC108348078 | 203 | 7.964892012 | | 38 | 1.507018602 | 5.285198206 | 2.401957577 |
| Adamts19 | 64 | 2.511098959 | | 12 | 0.475900611 | 5.276519719 | 2.399586672 |
| LOC100911769 | 165 | 6.473927004 | | 32 | 1.269068296 | 5.101322776 | 2.350871387 |
| Scnn1d | 20 | 0.784718425 | | 4 | 0.158633537 | 4.946737237 | 2.306477268 |
| Cyp2j4 | 10 | 0.392359212 | | 2 | 0.079316769 | 4.946737237 | 2.306477268 |
| RGD1561572 | 10 | 0.392359212 | | 2 | 0.079316769 | 4.946737237 | 2.306477268 |
| Spdya | 10 | 0.392359212 | | 2 | 0.079316769 | 4.946737237 | 2.306477268 |
| Tex26 | 10 | 0.392359212 | | 2 | 0.079316769 | 4.946737237 | 2.306477268 |
| Ak9 | 5 | 0.196179606 | | 1 | 0.039658384 | 4.946737237 | 2.306477268 |
| Anks4b | 5 | 0.196179606 | | 1 | 0.039658384 | 4.946737237 | 2.306477268 |
| Csrnp3 | 5 | 0.196179606 | | 1 | 0.039658384 | 4.946737237 | 2.306477268 |
| Fabp12 | 5 | 0.196179606 | | 1 | 0.039658384 | 4.946737237 | 2.306477268 |
| Hormad2 | 5 | 0.196179606 | | 1 | 0.039658384 | 4.946737237 | 2.306477268 |
| LOC102549226 | 5 | 0.196179606 | | 1 | 0.039658384 | 4.946737237 | 2.306477268 |
| LOC102551647 | 5 | 0.196179606 | | 1 | 0.039658384 | 4.946737237 | 2.306477268 |
| LOC102552714 | 5 | 0.196179606 | | 1 | 0.039658384 | 4.946737237 | 2.306477268 |
| LOC102554658 | 5 | 0.196179606 | | 1 | 0.039658384 | 4.946737237 | 2.306477268 |
| LOC102555078 | 5 | 0.196179606 | | 1 | 0.039658384 | 4.946737237 | 2.306477268 |
| LOC108348879 | 5 | 0.196179606 | | 1 | 0.039658384 | 4.946737237 | 2.306477268 |
| LOC108350355 | 5 | 0.196179606 | | 1 | 0.039658384 | 4.946737237 | 2.306477268 |
| LOC108350810 | 5 | 0.196179606 | | 1 | 0.039658384 | 4.946737237 | 2.306477268 |
| LOC108352033 | 5 | 0.196179606 | | 1 | 0.039658384 | 4.946737237 | 2.306477268 |
| LOC680341 | 5 | 0.196179606 | | 1 | 0.039658384 | 4.946737237 | 2.306477268 |
| Lax1 | 5 | 0.196179606 | | 1 | 0.039658384 | 4.946737237 | 2.306477268 |
| Ldlrad2 | 5 | 0.196179606 | | 1 | 0.039658384 | 4.946737237 | 2.306477268 |
| Lexm | 5 | 0.196179606 | | 1 | 0.039658384 | 4.946737237 | 2.306477268 |
| Lrrc4b | 5 | 0.196179606 | | 1 | 0.039658384 | 4.946737237 | 2.306477268 |
| Lsmem1 | 5 | 0.196179606 | | 1 | 0.039658384 | 4.946737237 | 2.306477268 |
| Mir152 | 5 | 0.196179606 | | 1 | 0.039658384 | 4.946737237 | 2.306477268 |
| Mir331 | 5 | 0.196179606 | | 1 | 0.039658384 | 4.946737237 | 2.306477268 |
| RGD1561102 | 5 | 0.196179606 | | 1 | 0.039658384 | 4.946737237 | 2.306477268 |
| RGD1561333 | 5 | 0.196179606 | | 1 | 0.039658384 | 4.946737237 | 2.306477268 |
| Rpl21-ps1 | 5 | 0.196179606 | | 1 | 0.039658384 | 4.946737237 | 2.306477268 |
| Scml4 | 5 | 0.196179606 | | 1 | 0.039658384 | 4.946737237 | 2.306477268 |
| Slc44a4 | 5 | 0.196179606 | | 1 | 0.039658384 | 4.946737237 | 2.306477268 |
| Syn3 | 5 | 0.196179606 | | 1 | 0.039658384 | 4.946737237 | 2.306477268 |
| Trmt61a | 5 | 0.196179606 | | 1 | 0.039658384 | 4.946737237 | 2.306477268 |
| Tldc2 | 14 | 0.549302897 | | 3 | 0.118975153 | 4.616954754 | 2.206941594 |
| Ttc36 | 14 | 0.549302897 | | 3 | 0.118975153 | 4.616954754 | 2.206941594 |
| Nefm | 27 | 1.059369873 | | 6 | 0.237950306 | 4.452063513 | 2.154474174 |
| LOC100362023 | 9 | 0.353123291 | | 2 | 0.079316769 | 4.452063513 | 2.154474174 |
| LOC108353319 | 9 | 0.353123291 | | 2 | 0.079316769 | 4.452063513 | 2.154474174 |
| Lrrc10b | 9 | 0.353123291 | | 2 | 0.079316769 | 4.452063513 | 2.154474174 |
| Rbp4 | 9 | 0.353123291 | | 2 | 0.079316769 | 4.452063513 | 2.154474174 |
| Scg5 | 9 | 0.353123291 | | 2 | 0.079316769 | 4.452063513 | 2.154474174 |
| Tmem212 | 9 | 0.353123291 | | 2 | 0.079316769 | 4.452063513 | 2.154474174 |
| Col1a2 | 151865 | 5958.563179 | | 34494 | 1367.976307 | 4.355750278 | 2.122921245 |
| Sparcl1 | 44 | 1.726380535 | | 10 | 0.396583843 | 4.353128768 | 2.122052697 |
| Hey1 | 78 | 3.060401857 | | 18 | 0.713850917 | 4.287172272 | 2.10002639 |
| Fam229a | 13 | 0.510066976 | | 3 | 0.118975153 | 4.287172272 | 2.10002639 |
| LOC100363500 | 13 | 0.510066976 | | 3 | 0.118975153 | 4.287172272 | 2.10002639 |
| Mall | 38 | 1.490965007 | | 9 | 0.356925458 | 4.177244778 | 2.062551685 |
| LOC102556397 | 50 | 1.961796062 | | 12 | 0.475900611 | 4.122281031 | 2.043442862 |
| Zfp74 | 50 | 1.961796062 | | 12 | 0.475900611 | 4.122281031 | 2.043442862 |
| LOC365985 | 58 | 2.275683432 | | 14 | 0.55521738 | 4.098725139 | 2.035175246 |
| Xirp1 | 37 | 1.451729086 | | 9 | 0.356925458 | 4.067317284 | 2.024077537 |
| Ndufa1 | 2 | 0.078471842 | | 558 | 22.12937842 | 0.003546048 | -8.139572139 |
| Nsun4 | 1 | 0.039235921 | | 112 | 4.441739038 | 0.008833459 | -6.822805749 |
| Tnip3 | 1 | 0.039235921 | | 104 | 4.124471963 | 0.009512956 | -6.715890545 |
| Ccdc142 | 0 | 0.019617961 | | 48 | 1.903602445 | 0.010305703 | -6.600413328 |
| LOC103694867 | 3 | 0.117707764 | | 192 | 7.614409779 | 0.015458554 | -6.015450827 |
| Nupr1 | 77 | 3.021165935 | | 4414 | 175.0521081 | 0.017258666 | -5.856535201 |
| Ccl7 | 46 | 1.804852377 | | 2210 | 87.64502922 | 0.020592752 | -5.601719525 |
| Ccl2 | 155 | 6.081567792 | | 6413 | 254.3292183 | 0.023912187 | -5.386110114 |
| Exoc7 | 9 | 0.353123291 | | 361 | 14.31667672 | 0.024665172 | -5.341380853 |
| Tnfrsf9 | 0 | 0.019617961 | | 20 | 0.793167685 | 0.024733686 | -5.337378922 |
| LOC103690068 | 16 | 0.62777474 | | 638 | 25.30204916 | 0.024811221 | -5.332863441 |
| Cxcl6 | 0 | 0.019617961 | | 19 | 0.753509301 | 0.026035459 | -5.263378341 |
| Ass1 | 1 | 0.039235921 | | 36 | 1.427701834 | 0.027481874 | -5.185375829 |
| LOC100909392 | 5 | 0.196179606 | | 177 | 7.019534015 | 0.027947668 | -5.161128282 |
| LOC100911130 | 4 | 0.156943685 | | 138 | 5.472857028 | 0.028676738 | -5.123975284 |
| LOC100912538 | 4 | 0.156943685 | | 130 | 5.155589954 | 0.03044146 | -5.03781864 |
| LOC103694380 | 0 | 0.019617961 | | 15 | 0.594875764 | 0.032978248 | -4.922341423 |
| LOC100910882 | 23 | 0.902426189 | | 662 | 26.25385038 | 0.034373099 | -4.862576278 |
| MGC94199 | 5 | 0.196179606 | | 142 | 5.631490566 | 0.034836178 | -4.843269852 |
| Capns1 | 33 | 1.294785401 | | 898 | 35.61322907 | 0.036356866 | -4.781628342 |
| Bag5 | 3 | 0.117707764 | | 81 | 3.212329125 | 0.036642498 | -4.770338329 |
| Fam3b | 0 | 0.019617961 | | 13 | 0.515558995 | 0.038051825 | -4.715890545 |
| Cxcl3 | 1 | 0.039235921 | | 24 | 0.951801222 | 0.04122281 | -4.600413328 |
| Mx1 | 0 | 0.019617961 | | 12 | 0.475900611 | 0.04122281 | -4.600413328 |
| LOC100911374 | 9 | 0.353123291 | | 198 | 7.852360084 | 0.044970339 | -4.474882446 |
| Ms4a6bl | 0 | 0.019617961 | | 11 | 0.436242227 | 0.044970339 | -4.474882446 |
| LOC100911928 | 9 | 0.353123291 | | 194 | 7.693726547 | 0.045897562 | -4.445438668 |
| Rmnd5b | 6 | 0.235415527 | | 128 | 5.076273186 | 0.046375662 | -4.430488326 |
| Hsp90aa1 | 52 | 2.040267904 | | 1105 | 43.82251461 | 0.046557527 | -4.424841763 |
| LOC100909595 | 12 | 0.470831055 | | 248 | 9.835279298 | 0.047871651 | -4.384684637 |
| LOC102547693 | 0 | 0.019617961 | | 10 | 0.396583843 | 0.049467372 | -4.337378922 |
| Wt1 | 0 | 0.019617961 | | 10 | 0.396583843 | 0.049467372 | -4.337378922 |
| Echs1 | 22 | 0.863190267 | | 439 | 17.41003069 | 0.049580054 | -4.334096338 |
| LOC108348055 | 4 | 0.156943685 | | 75 | 2.97437882 | 0.052765197 | -4.244269518 |
| Aldh3b2 | 0 | 0.019617961 | | 9 | 0.356925458 | 0.054963747 | -4.185375829 |
| Ccl1 | 0 | 0.019617961 | | 9 | 0.356925458 | 0.054963747 | -4.185375829 |
| Il1b | 0 | 0.019617961 | | 9 | 0.356925458 | 0.054963747 | -4.185375829 |
| Slc13a3 | 0 | 0.019617961 | | 9 | 0.356925458 | 0.054963747 | -4.185375829 |
| LOC685048 | 0 | 0.019617961 | | 8 | 0.317267074 | 0.061834215 | -4.015450827 |
| Sbk1 | 0 | 0.019617961 | | 8 | 0.317267074 | 0.061834215 | -4.015450827 |
| Aldh18a1 | 43 | 1.687144613 | | 653 | 25.89692492 | 0.065148454 | -3.940125254 |
| Prph | 1 | 0.039235921 | | 14 | 0.55521738 | 0.070667675 | -3.822805749 |
| Acpp | 0 | 0.019617961 | | 7 | 0.27760869 | 0.070667675 | -3.822805749 |
| LOC100363557 | 0 | 0.019617961 | | 7 | 0.27760869 | 0.070667675 | -3.822805749 |
| LOC100910636 | 0 | 0.019617961 | | 7 | 0.27760869 | 0.070667675 | -3.822805749 |
| Pax6 | 0 | 0.019617961 | | 7 | 0.27760869 | 0.070667675 | -3.822805749 |
| Trex2 | 0 | 0.019617961 | | 7 | 0.27760869 | 0.070667675 | -3.822805749 |
| Zfp551 | 0 | 0.019617961 | | 7 | 0.27760869 | 0.070667675 | -3.822805749 |
| Rbp1 | 7 | 0.274651449 | | 97 | 3.846863274 | 0.071396208 | -3.808008747 |
| LOC100911994 | 5 | 0.196179606 | | 68 | 2.69677013 | 0.072746136 | -3.780985573 |
| Vwa5a | 20 | 0.784718425 | | 259 | 10.27152152 | 0.076397486 | -3.71033102 |
| LOC103690018 | 16 | 0.62777474 | | 207 | 8.209285543 | 0.0764713 | -3.708937785 |
| LOC102554336 | 1 | 0.039235921 | | 12 | 0.475900611 | 0.082445621 | -3.600413328 |
| Cadm3 | 0 | 0.019617961 | | 6 | 0.237950306 | 0.082445621 | -3.600413328 |
| Ccl9 | 0 | 0.019617961 | | 6 | 0.237950306 | 0.082445621 | -3.600413328 |
| Cfap126 | 0 | 0.019617961 | | 6 | 0.237950306 | 0.082445621 | -3.600413328 |
| Cgn | 0 | 0.019617961 | | 6 | 0.237950306 | 0.082445621 | -3.600413328 |
| Dlx4 | 0 | 0.019617961 | | 6 | 0.237950306 | 0.082445621 | -3.600413328 |
| Dsc3 | 0 | 0.019617961 | | 6 | 0.237950306 | 0.082445621 | -3.600413328 |
| Fam129c | 0 | 0.019617961 | | 6 | 0.237950306 | 0.082445621 | -3.600413328 |
| Fam71d | 0 | 0.019617961 | | 6 | 0.237950306 | 0.082445621 | -3.600413328 |
| Fam83g | 0 | 0.019617961 | | 6 | 0.237950306 | 0.082445621 | -3.600413328 |
| LOC108348394 | 0 | 0.019617961 | | 6 | 0.237950306 | 0.082445621 | -3.600413328 |
| LOC108351762 | 0 | 0.019617961 | | 6 | 0.237950306 | 0.082445621 | -3.600413328 |
| LOC689229 | 0 | 0.019617961 | | 6 | 0.237950306 | 0.082445621 | -3.600413328 |
| Mylk4 | 0 | 0.019617961 | | 6 | 0.237950306 | 0.082445621 | -3.600413328 |
| Sox18 | 0 | 0.019617961 | | 6 | 0.237950306 | 0.082445621 | -3.600413328 |
| Adamts8 | 5 | 0.196179606 | | 58 | 2.300186287 | 0.085288573 | -3.551503727 |
| Cxcl1 | 11 | 0.431595134 | | 125 | 4.957298033 | 0.087062575 | -3.521803493 |
| Tspan4 | 10 | 0.392359212 | | 110 | 4.362422269 | 0.089940677 | -3.474882446 |
| LOC100911727 | 9 | 0.353123291 | | 99 | 3.926180042 | 0.089940677 | -3.474882446 |
| Catsper4 | 1 | 0.039235921 | | 11 | 0.436242227 | 0.089940677 | -3.474882446 |
| Ccl12 | 1 | 0.039235921 | | 11 | 0.436242227 | 0.089940677 | -3.474882446 |
| LOC108350598 | 1 | 0.039235921 | | 11 | 0.436242227 | 0.089940677 | -3.474882446 |
| LOC24906 | 1 | 0.039235921 | | 11 | 0.436242227 | 0.089940677 | -3.474882446 |
| LOC680375 | 1 | 0.039235921 | | 11 | 0.436242227 | 0.089940677 | -3.474882446 |
| Mast4 | 24 | 0.94166211 | | 261 | 10.35083829 | 0.090974478 | -3.458394323 |
| Igsf7 | 1 | 0.039235921 | | 10 | 0.396583843 | 0.098934745 | -3.337378922 |
| LOC100361645 | 1 | 0.039235921 | | 10 | 0.396583843 | 0.098934745 | -3.337378922 |
| LOC103692142 | 1 | 0.039235921 | | 10 | 0.396583843 | 0.098934745 | -3.337378922 |
| Mmp10 | 1 | 0.039235921 | | 10 | 0.396583843 | 0.098934745 | -3.337378922 |
| Akap14 | 0 | 0.019617961 | | 5 | 0.198291921 | 0.098934745 | -3.337378922 |
| Arg2 | 0 | 0.019617961 | | 5 | 0.198291921 | 0.098934745 | -3.337378922 |
| Bco1 | 0 | 0.019617961 | | 5 | 0.198291921 | 0.098934745 | -3.337378922 |
| Cxcr5 | 0 | 0.019617961 | | 5 | 0.198291921 | 0.098934745 | -3.337378922 |
| Gzmbl2 | 0 | 0.019617961 | | 5 | 0.198291921 | 0.098934745 | -3.337378922 |
| Kcnh6 | 0 | 0.019617961 | | 5 | 0.198291921 | 0.098934745 | -3.337378922 |
| LOC100911190 | 0 | 0.019617961 | | 5 | 0.198291921 | 0.098934745 | -3.337378922 |
| LOC100911486 | 0 | 0.019617961 | | 5 | 0.198291921 | 0.098934745 | -3.337378922 |
| LOC102547504 | 0 | 0.019617961 | | 5 | 0.198291921 | 0.098934745 | -3.337378922 |
| LOC103694166 | 0 | 0.019617961 | | 5 | 0.198291921 | 0.098934745 | -3.337378922 |
| LOC103695208 | 0 | 0.019617961 | | 5 | 0.198291921 | 0.098934745 | -3.337378922 |
| LOC108350553 | 0 | 0.019617961 | | 5 | 0.198291921 | 0.098934745 | -3.337378922 |
| LOC108353237 | 0 | 0.019617961 | | 5 | 0.198291921 | 0.098934745 | -3.337378922 |
| LOC367856 | 0 | 0.019617961 | | 5 | 0.198291921 | 0.098934745 | -3.337378922 |
| LOC685511 | 0 | 0.019617961 | | 5 | 0.198291921 | 0.098934745 | -3.337378922 |
| Lama1 | 0 | 0.019617961 | | 5 | 0.198291921 | 0.098934745 | -3.337378922 |
| Lingo3 | 0 | 0.019617961 | | 5 | 0.198291921 | 0.098934745 | -3.337378922 |
| Mir770 | 0 | 0.019617961 | | 5 | 0.198291921 | 0.098934745 | -3.337378922 |
| Mpa2l | 0 | 0.019617961 | | 5 | 0.198291921 | 0.098934745 | -3.337378922 |
| Pdc | 0 | 0.019617961 | | 5 | 0.198291921 | 0.098934745 | -3.337378922 |
| RGD1306782 | 0 | 0.019617961 | | 5 | 0.198291921 | 0.098934745 | -3.337378922 |
| RGD1564854 | 0 | 0.019617961 | | 5 | 0.198291921 | 0.098934745 | -3.337378922 |
| Rps15al2 | 0 | 0.019617961 | | 5 | 0.198291921 | 0.098934745 | -3.337378922 |
| Tnfrsf19 | 0 | 0.019617961 | | 5 | 0.198291921 | 0.098934745 | -3.337378922 |
| Tspan1 | 0 | 0.019617961 | | 5 | 0.198291921 | 0.098934745 | -3.337378922 |
| Zfp536 | 0 | 0.019617961 | | 5 | 0.198291921 | 0.098934745 | -3.337378922 |
| Slc47a1 | 11 | 0.431595134 | | 109 | 4.322763885 | 0.099842403 | -3.324203533 |
| LOC103689941 | 5 | 0.196179606 | | 47 | 1.86394406 | 0.105249728 | -3.248111584 |
| LOC103690028 | 3 | 0.117707764 | | 27 | 1.070776375 | 0.109927494 | -3.185375829 |
| Alox15 | 1 | 0.039235921 | | 9 | 0.356925458 | 0.109927494 | -3.185375829 |
| Cdhr4 | 1 | 0.039235921 | | 9 | 0.356925458 | 0.109927494 | -3.185375829 |
| LOC100910497 | 1 | 0.039235921 | | 9 | 0.356925458 | 0.109927494 | -3.185375829 |
| LOC102555659 | 1 | 0.039235921 | | 9 | 0.356925458 | 0.109927494 | -3.185375829 |
| LOC691320 | 1 | 0.039235921 | | 9 | 0.356925458 | 0.109927494 | -3.185375829 |
| Trem3 | 1 | 0.039235921 | | 9 | 0.356925458 | 0.109927494 | -3.185375829 |
| Slc7a4 | 2 | 0.078471842 | | 17 | 0.674192532 | 0.116393817 | -3.102913668 |
| Zmynd15 | 2 | 0.078471842 | | 17 | 0.674192532 | 0.116393817 | -3.102913668 |
| Frg1l1 | 7 | 0.274651449 | | 59 | 2.339844672 | 0.117380206 | -3.090738954 |
| Lrrn4 | 8 | 0.31388737 | | 66 | 2.617453361 | 0.119920903 | -3.059844946 |
| RT1-Ba | 4 | 0.156943685 | | 33 | 1.308726681 | 0.119920903 | -3.059844946 |
| RT1-CE7 | 8 | 0.31388737 | | 65 | 2.577794977 | 0.12176584 | -3.03781864 |
| Agap2 | 3 | 0.117707764 | | 24 | 0.951801222 | 0.123668431 | -3.015450827 |
| Ankrd33b | 1 | 0.039235921 | | 8 | 0.317267074 | 0.123668431 | -3.015450827 |
| Cfap57 | 1 | 0.039235921 | | 8 | 0.317267074 | 0.123668431 | -3.015450827 |
| Dnaaf1 | 1 | 0.039235921 | | 8 | 0.317267074 | 0.123668431 | -3.015450827 |
| Fmr1nb | 1 | 0.039235921 | | 8 | 0.317267074 | 0.123668431 | -3.015450827 |
| LOC103690016 | 1 | 0.039235921 | | 8 | 0.317267074 | 0.123668431 | -3.015450827 |
| LOC103690020 | 1 | 0.039235921 | | 8 | 0.317267074 | 0.123668431 | -3.015450827 |
| LOC309220 | 1 | 0.039235921 | | 8 | 0.317267074 | 0.123668431 | -3.015450827 |
| LOC498276 | 1 | 0.039235921 | | 8 | 0.317267074 | 0.123668431 | -3.015450827 |
| Rps9 | 1 | 0.039235921 | | 8 | 0.317267074 | 0.123668431 | -3.015450827 |
| LOC103690017 | 9 | 0.353123291 | | 71 | 2.815745283 | 0.12541024 | -2.995272945 |
| LOC100912585 | 21 | 0.823954346 | | 165 | 6.543633404 | 0.125916948 | -2.989455619 |
| Neurl3 | 8 | 0.31388737 | | 62 | 2.458819824 | 0.127657735 | -2.969647137 |
| Stap2 | 6 | 0.235415527 | | 46 | 1.824285676 | 0.129045319 | -2.954050282 |
| C4b | 2 | 0.078471842 | | 15 | 0.594875764 | 0.131912993 | -2.922341423 |
| C1s | 149 | 5.846152265 | | 1092 | 43.30695562 | 0.134993379 | -2.889039448 |
| LOC103693015 | 20 | 0.784718425 | | 144 | 5.710807334 | 0.137409368 | -2.863447734 |
| Trim30 | 5 | 0.196179606 | | 36 | 1.427701834 | 0.137409368 | -2.863447734 |
| Cd36 | 4 | 0.156943685 | | 28 | 1.110434759 | 0.14133535 | -2.822805749 |
| Pstpip2 | 2 | 0.078471842 | | 14 | 0.55521738 | 0.14133535 | -2.822805749 |
| Ace | 1 | 0.039235921 | | 7 | 0.27760869 | 0.14133535 | -2.822805749 |
| Atp6v0d2 | 1 | 0.039235921 | | 7 | 0.27760869 | 0.14133535 | -2.822805749 |
| B4galnt3 | 1 | 0.039235921 | | 7 | 0.27760869 | 0.14133535 | -2.822805749 |
| Il1a | 1 | 0.039235921 | | 7 | 0.27760869 | 0.14133535 | -2.822805749 |
| LOC291138 | 1 | 0.039235921 | | 7 | 0.27760869 | 0.14133535 | -2.822805749 |
| Sfrp5 | 1 | 0.039235921 | | 7 | 0.27760869 | 0.14133535 | -2.822805749 |
| Zbtb33 | 1 | 0.039235921 | | 7 | 0.27760869 | 0.14133535 | -2.822805749 |
| Scube3 | 1751 | 68.70209809 | | 11938 | 473.4417913 | 0.145112027 | -2.784760997 |
| LOC108348052 | 11 | 0.431595134 | | 74 | 2.934720436 | 0.147065161 | -2.765472574 |
| Mmp9 | 36 | 1.412493165 | | 242 | 9.597328992 | 0.147175653 | -2.764389063 |
| Rxfp3 | 3 | 0.117707764 | | 20 | 0.793167685 | 0.148402117 | -2.752416421 |
| Cdh23 | 2 | 0.078471842 | | 13 | 0.515558995 | 0.1522073 | -2.715890545 |
| Dcaf12 | 2 | 0.078471842 | | 13 | 0.515558995 | 0.1522073 | -2.715890545 |
| LOC100361713 | 2 | 0.078471842 | | 13 | 0.515558995 | 0.1522073 | -2.715890545 |
| LOC689407 | 2 | 0.078471842 | | 13 | 0.515558995 | 0.1522073 | -2.715890545 |
| Scin | 2 | 0.078471842 | | 13 | 0.515558995 | 0.1522073 | -2.715890545 |
| Cytip | 6 | 0.235415527 | | 38 | 1.507018602 | 0.156212755 | -2.67841584 |
| Slc25a22 | 3 | 0.117707764 | | 19 | 0.753509301 | 0.156212755 | -2.67841584 |
| LOC108349745 | 7 | 0.274651449 | | 44 | 1.744968908 | 0.157396185 | -2.667527524 |
| Ogn | 194 | 7.61176872 | | 1204 | 47.74869465 | 0.159413127 | -2.649157662 |
| LOC108348122 | 91 | 3.570468833 | | 555 | 22.01040327 | 0.162217329 | -2.624000148 |
| LOC681325 | 4 | 0.156943685 | | 24 | 0.951801222 | 0.164891241 | -2.600413328 |
| LOC499229 | 3 | 0.117707764 | | 18 | 0.713850917 | 0.164891241 | -2.600413328 |
| Pnpla3 | 2 | 0.078471842 | | 12 | 0.475900611 | 0.164891241 | -2.600413328 |
| Btn1a1 | 1 | 0.039235921 | | 6 | 0.237950306 | 0.164891241 | -2.600413328 |
| Cmtm5 | 1 | 0.039235921 | | 6 | 0.237950306 | 0.164891241 | -2.600413328 |
| Gng11 | 1 | 0.039235921 | | 6 | 0.237950306 | 0.164891241 | -2.600413328 |
| Klra1 | 1 | 0.039235921 | | 6 | 0.237950306 | 0.164891241 | -2.600413328 |
| LOC100911537 | 1 | 0.039235921 | | 6 | 0.237950306 | 0.164891241 | -2.600413328 |
| LOC108348127 | 1 | 0.039235921 | | 6 | 0.237950306 | 0.164891241 | -2.600413328 |
| LOC108352377 | 1 | 0.039235921 | | 6 | 0.237950306 | 0.164891241 | -2.600413328 |
| Mcemp1 | 1 | 0.039235921 | | 6 | 0.237950306 | 0.164891241 | -2.600413328 |
| Meig1 | 1 | 0.039235921 | | 6 | 0.237950306 | 0.164891241 | -2.600413328 |
| RGD1563492 | 1 | 0.039235921 | | 6 | 0.237950306 | 0.164891241 | -2.600413328 |
| Ret | 1 | 0.039235921 | | 6 | 0.237950306 | 0.164891241 | -2.600413328 |
| Snph | 1 | 0.039235921 | | 6 | 0.237950306 | 0.164891241 | -2.600413328 |
| Sult1a1 | 1 | 0.039235921 | | 6 | 0.237950306 | 0.164891241 | -2.600413328 |
| MGC105649 | 10 | 0.392359212 | | 59 | 2.339844672 | 0.167686008 | -2.576165782 |
| LOC100910438 | 14 | 0.549302897 | | 81 | 3.212329125 | 0.170998324 | -2.547945908 |
| Cyp4f18 | 3 | 0.117707764 | | 17 | 0.674192532 | 0.174590726 | -2.517951168 |
| Map3k7 | 68 | 2.668042644 | | 384 | 15.22881956 | 0.175196944 | -2.512950487 |
| C3 | 15 | 0.588538819 | | 84 | 3.331304278 | 0.176669187 | -2.500877654 |
| Slamf8 | 10 | 0.392359212 | | 55 | 2.181211135 | 0.179881354 | -2.474882446 |
| LOC108348145 | 4 | 0.156943685 | | 22 | 0.872484454 | 0.179881354 | -2.474882446 |
| Ccl20 | 2 | 0.078471842 | | 11 | 0.436242227 | 0.179881354 | -2.474882446 |
| Ctla2a | 2 | 0.078471842 | | 11 | 0.436242227 | 0.179881354 | -2.474882446 |
| Galnt13 | 2 | 0.078471842 | | 11 | 0.436242227 | 0.179881354 | -2.474882446 |
| Tmem63c | 2 | 0.078471842 | | 11 | 0.436242227 | 0.179881354 | -2.474882446 |
| Bcl3 | 46 | 1.804852377 | | 250 | 9.914596066 | 0.18203993 | -2.457673156 |
| Wnt2b | 5 | 0.196179606 | | 27 | 1.070776375 | 0.18321249 | -2.448410234 |
| LOC108348197 | 3 | 0.117707764 | | 16 | 0.634534148 | 0.185502646 | -2.430488326 |
| Mir223 | 3 | 0.117707764 | | 16 | 0.634534148 | 0.185502646 | -2.430488326 |
| Ccnj | 4 | 0.156943685 | | 21 | 0.83282607 | 0.188447133 | -2.40776825 |
| Rufy2 | 6 | 0.235415527 | | 31 | 1.229409912 | 0.191486603 | -2.384684637 |
| LOC100360647 | 5 | 0.196179606 | | 25 | 0.991459607 | 0.197869489 | -2.337378922 |
| Ptges | 3 | 0.117707764 | | 15 | 0.594875764 | 0.197869489 | -2.337378922 |
| Apln | 2 | 0.078471842 | | 10 | 0.396583843 | 0.197869489 | -2.337378922 |
| Clec2d2 | 2 | 0.078471842 | | 10 | 0.396583843 | 0.197869489 | -2.337378922 |
| LOC102552645 | 2 | 0.078471842 | | 10 | 0.396583843 | 0.197869489 | -2.337378922 |
| LOC103695344 | 2 | 0.078471842 | | 10 | 0.396583843 | 0.197869489 | -2.337378922 |
| LOC685279 | 2 | 0.078471842 | | 10 | 0.396583843 | 0.197869489 | -2.337378922 |
| Lamc3 | 2 | 0.078471842 | | 10 | 0.396583843 | 0.197869489 | -2.337378922 |
| Pkd2l2 | 2 | 0.078471842 | | 10 | 0.396583843 | 0.197869489 | -2.337378922 |
| Tmem156 | 2 | 0.078471842 | | 10 | 0.396583843 | 0.197869489 | -2.337378922 |
| Adgrv1 | 1 | 0.039235921 | | 5 | 0.198291921 | 0.197869489 | -2.337378922 |
| C1qtnf9 | 1 | 0.039235921 | | 5 | 0.198291921 | 0.197869489 | -2.337378922 |
| Ccl6 | 1 | 0.039235921 | | 5 | 0.198291921 | 0.197869489 | -2.337378922 |
| Erp27 | 1 | 0.039235921 | | 5 | 0.198291921 | 0.197869489 | -2.337378922 |
| Gpr4 | 1 | 0.039235921 | | 5 | 0.198291921 | 0.197869489 | -2.337378922 |
| Grm4 | 1 | 0.039235921 | | 5 | 0.198291921 | 0.197869489 | -2.337378922 |
| Hils1 | 1 | 0.039235921 | | 5 | 0.198291921 | 0.197869489 | -2.337378922 |
| Hist3h2ba | 1 | 0.039235921 | | 5 | 0.198291921 | 0.197869489 | -2.337378922 |
| Hprt1 | 1 | 0.039235921 | | 5 | 0.198291921 | 0.197869489 | -2.337378922 |
| Jph3 | 1 | 0.039235921 | | 5 | 0.198291921 | 0.197869489 | -2.337378922 |
| Klhl34 | 1 | 0.039235921 | | 5 | 0.198291921 | 0.197869489 | -2.337378922 |
| LOC100359928 | 1 | 0.039235921 | | 5 | 0.198291921 | 0.197869489 | -2.337378922 |
| LOC100361854 | 1 | 0.039235921 | | 5 | 0.198291921 | 0.197869489 | -2.337378922 |
| LOC100912642 | 1 | 0.039235921 | | 5 | 0.198291921 | 0.197869489 | -2.337378922 |
| LOC102548396 | 1 | 0.039235921 | | 5 | 0.198291921 | 0.197869489 | -2.337378922 |
| LOC102550219 | 1 | 0.039235921 | | 5 | 0.198291921 | 0.197869489 | -2.337378922 |
| LOC102554277 | 1 | 0.039235921 | | 5 | 0.198291921 | 0.197869489 | -2.337378922 |
| LOC689590 | 1 | 0.039235921 | | 5 | 0.198291921 | 0.197869489 | -2.337378922 |
| LOC690857 | 1 | 0.039235921 | | 5 | 0.198291921 | 0.197869489 | -2.337378922 |
| Peli1-ps1 | 1 | 0.039235921 | | 5 | 0.198291921 | 0.197869489 | -2.337378922 |
| RGD1560394 | 1 | 0.039235921 | | 5 | 0.198291921 | 0.197869489 | -2.337378922 |
| RGD1560554 | 1 | 0.039235921 | | 5 | 0.198291921 | 0.197869489 | -2.337378922 |
| RGD1563705 | 1 | 0.039235921 | | 5 | 0.198291921 | 0.197869489 | -2.337378922 |
| Rhbg | 1 | 0.039235921 | | 5 | 0.198291921 | 0.197869489 | -2.337378922 |
| Sntg2 | 1 | 0.039235921 | | 5 | 0.198291921 | 0.197869489 | -2.337378922 |
| Tvp23a | 1 | 0.039235921 | | 5 | 0.198291921 | 0.197869489 | -2.337378922 |
| Trem1 | 9 | 0.353123291 | | 44 | 1.744968908 | 0.202366523 | -2.304957444 |
| Cd74 | 127 | 4.982961997 | | 613 | 24.31058955 | 0.204970841 | -2.286509404 |
| RT1-Bb | 5 | 0.196179606 | | 24 | 0.951801222 | 0.206114052 | -2.278485233 |
| LOC100911779 | 25 | 0.980898031 | | 119 | 4.719347727 | 0.207846102 | -2.266412401 |
| LOC688442 | 8 | 0.31388737 | | 38 | 1.507018602 | 0.208283673 | -2.263378341 |
| Ccr5 | 12 | 0.470831055 | | 56 | 2.220869519 | 0.212003024 | -2.237843248 |
| LOC685067 | 3 | 0.117707764 | | 14 | 0.55521738 | 0.212003024 | -2.237843248 |
| Tmco3 | 3 | 0.117707764 | | 14 | 0.55521738 | 0.212003024 | -2.237843248 |
| B3gnt8 | 4 | 0.156943685 | | 18 | 0.713850917 | 0.219854988 | -2.185375829 |
| Hoxb7 | 4 | 0.156943685 | | 18 | 0.713850917 | 0.219854988 | -2.185375829 |
| Lilrb3a | 4 | 0.156943685 | | 18 | 0.713850917 | 0.219854988 | -2.185375829 |
| Ccdc116 | 2 | 0.078471842 | | 9 | 0.356925458 | 0.219854988 | -2.185375829 |
| Clec3b | 2 | 0.078471842 | | 9 | 0.356925458 | 0.219854988 | -2.185375829 |
| Klk4 | 2 | 0.078471842 | | 9 | 0.356925458 | 0.219854988 | -2.185375829 |
| Ppl | 2 | 0.078471842 | | 9 | 0.356925458 | 0.219854988 | -2.185375829 |
| Slc12a3 | 2 | 0.078471842 | | 9 | 0.356925458 | 0.219854988 | -2.185375829 |
| Tnfaip3 | 168 | 6.591634768 | | 752 | 29.82310497 | 0.22102443 | -2.177722256 |
| Pram1 | 9 | 0.353123291 | | 40 | 1.586335371 | 0.222603176 | -2.167453921 |
| Eno4 | 7 | 0.274651449 | | 31 | 1.229409912 | 0.223401037 | -2.162292215 |
| Aim1 | 5 | 0.196179606 | | 22 | 0.872484454 | 0.224851693 | -2.152954351 |
| LOC103693683 | 5 | 0.196179606 | | 22 | 0.872484454 | 0.224851693 | -2.152954351 |
| Prkcq | 8 | 0.31388737 | | 35 | 1.388043449 | 0.226136559 | -2.144733844 |
| NEWGENE_621802 | 76 | 2.981930014 | | 331 | 13.12692519 | 0.227161347 | -2.13821072 |
| Pddc1 | 40 | 1.56943685 | | 174 | 6.900558862 | 0.227436195 | -2.136466228 |
| Kcnk5 | 3 | 0.117707764 | | 13 | 0.515558995 | 0.228310949 | -2.130928045 |
| LOC102546870 | 3 | 0.117707764 | | 13 | 0.515558995 | 0.228310949 | -2.130928045 |
| LOC102555727 | 29 | 1.137841716 | | 125 | 4.957298033 | 0.229528608 | -2.123254117 |
| Atp1a3 | 33 | 1.294785401 | | 141 | 5.591832181 | 0.231549403 | -2.11060806 |
| RGD1563601 | 4 | 0.156943685 | | 17 | 0.674192532 | 0.232787635 | -2.102913668 |
| Gpr84 | 12 | 0.470831055 | | 50 | 1.982919213 | 0.237443387 | -2.074344516 |
| Ch25h | 38 | 1.490965007 | | 157 | 6.226366329 | 0.239459892 | -2.062144063 |
| Marf1 | 39 | 1.530200928 | | 157 | 6.226366329 | 0.245761468 | -2.024669357 |
| Il18 | 11 | 0.431595134 | | 44 | 1.744968908 | 0.247336862 | -2.015450827 |
| L1cam | 7 | 0.274651449 | | 28 | 1.110434759 | 0.247336862 | -2.015450827 |
| LOC108349973 | 6 | 0.235415527 | | 24 | 0.951801222 | 0.247336862 | -2.015450827 |
| Oxtr | 5 | 0.196179606 | | 20 | 0.793167685 | 0.247336862 | -2.015450827 |
| RGD1565785 | 5 | 0.196179606 | | 20 | 0.793167685 | 0.247336862 | -2.015450827 |
| LOC102547064 | 4 | 0.156943685 | | 16 | 0.634534148 | 0.247336862 | -2.015450827 |
| Ncmap | 4 | 0.156943685 | | 16 | 0.634534148 | 0.247336862 | -2.015450827 |
| Tcp11 | 4 | 0.156943685 | | 16 | 0.634534148 | 0.247336862 | -2.015450827 |
| Cd40 | 3 | 0.117707764 | | 12 | 0.475900611 | 0.247336862 | -2.015450827 |
| Hrh1 | 3 | 0.117707764 | | 12 | 0.475900611 | 0.247336862 | -2.015450827 |
| Lrrn1 | 3 | 0.117707764 | | 12 | 0.475900611 | 0.247336862 | -2.015450827 |
| Mir339 | 3 | 0.117707764 | | 12 | 0.475900611 | 0.247336862 | -2.015450827 |
| Wfdc18 | 3 | 0.117707764 | | 12 | 0.475900611 | 0.247336862 | -2.015450827 |
| Bfsp2 | 2 | 0.078471842 | | 8 | 0.317267074 | 0.247336862 | -2.015450827 |
| Dmrt1 | 2 | 0.078471842 | | 8 | 0.317267074 | 0.247336862 | -2.015450827 |
| Hsd11b2 | 2 | 0.078471842 | | 8 | 0.317267074 | 0.247336862 | -2.015450827 |
| Kcnk4 | 2 | 0.078471842 | | 8 | 0.317267074 | 0.247336862 | -2.015450827 |
| LOC100909726 | 2 | 0.078471842 | | 8 | 0.317267074 | 0.247336862 | -2.015450827 |
| LOC680663 | 2 | 0.078471842 | | 8 | 0.317267074 | 0.247336862 | -2.015450827 |
| LOC689903 | 2 | 0.078471842 | | 8 | 0.317267074 | 0.247336862 | -2.015450827 |
| Meltf | 2 | 0.078471842 | | 8 | 0.317267074 | 0.247336862 | -2.015450827 |
| RGD1561147 | 2 | 0.078471842 | | 8 | 0.317267074 | 0.247336862 | -2.015450827 |
| RGD1563066 | 2 | 0.078471842 | | 8 | 0.317267074 | 0.247336862 | -2.015450827 |
| Slc17a6 | 2 | 0.078471842 | | 8 | 0.317267074 | 0.247336862 | -2.015450827 |
| Syt1 | 2 | 0.078471842 | | 8 | 0.317267074 | 0.247336862 | -2.015450827 |
| Als2cr12 | 1 | 0.039235921 | | 4 | 0.158633537 | 0.247336862 | -2.015450827 |
| Ccl11 | 1 | 0.039235921 | | 4 | 0.158633537 | 0.247336862 | -2.015450827 |
| Ccrl2 | 1 | 0.039235921 | | 4 | 0.158633537 | 0.247336862 | -2.015450827 |
| Cdh17 | 1 | 0.039235921 | | 4 | 0.158633537 | 0.247336862 | -2.015450827 |
| Cyp4b1 | 1 | 0.039235921 | | 4 | 0.158633537 | 0.247336862 | -2.015450827 |
| Etnk2 | 1 | 0.039235921 | | 4 | 0.158633537 | 0.247336862 | -2.015450827 |
| Fam19a2 | 1 | 0.039235921 | | 4 | 0.158633537 | 0.247336862 | -2.015450827 |
| Grik2 | 1 | 0.039235921 | | 4 | 0.158633537 | 0.247336862 | -2.015450827 |
| Gstm6 | 1 | 0.039235921 | | 4 | 0.158633537 | 0.247336862 | -2.015450827 |
| Htr1d | 1 | 0.039235921 | | 4 | 0.158633537 | 0.247336862 | -2.015450827 |
| Ikzf3 | 1 | 0.039235921 | | 4 | 0.158633537 | 0.247336862 | -2.015450827 |
| Itgb7 | 1 | 0.039235921 | | 4 | 0.158633537 | 0.247336862 | -2.015450827 |
| Kcnip4 | 1 | 0.039235921 | | 4 | 0.158633537 | 0.247336862 | -2.015450827 |
| LOC100362149 | 1 | 0.039235921 | | 4 | 0.158633537 | 0.247336862 | -2.015450827 |
| LOC100362611 | 1 | 0.039235921 | | 4 | 0.158633537 | 0.247336862 | -2.015450827 |
| LOC100363423 | 1 | 0.039235921 | | 4 | 0.158633537 | 0.247336862 | -2.015450827 |
| LOC100365062 | 1 | 0.039235921 | | 4 | 0.158633537 | 0.247336862 | -2.015450827 |
| LOC100909448 | 1 | 0.039235921 | | 4 | 0.158633537 | 0.247336862 | -2.015450827 |
| LOC100910117 | 1 | 0.039235921 | | 4 | 0.158633537 | 0.247336862 | -2.015450827 |
| LOC100911453 | 1 | 0.039235921 | | 4 | 0.158633537 | 0.247336862 | -2.015450827 |
| LOC100912436 | 1 | 0.039235921 | | 4 | 0.158633537 | 0.247336862 | -2.015450827 |
| LOC102547390 | 1 | 0.039235921 | | 4 | 0.158633537 | 0.247336862 | -2.015450827 |
| LOC102556082 | 1 | 0.039235921 | | 4 | 0.158633537 | 0.247336862 | -2.015450827 |
| LOC103691294 | 1 | 0.039235921 | | 4 | 0.158633537 | 0.247336862 | -2.015450827 |
| LOC103692165 | 1 | 0.039235921 | | 4 | 0.158633537 | 0.247336862 | -2.015450827 |
| LOC108348226 | 1 | 0.039235921 | | 4 | 0.158633537 | 0.247336862 | -2.015450827 |
| LOC362473 | 1 | 0.039235921 | | 4 | 0.158633537 | 0.247336862 | -2.015450827 |
| LOC365572 | 1 | 0.039235921 | | 4 | 0.158633537 | 0.247336862 | -2.015450827 |
| Lmtk3 | 1 | 0.039235921 | | 4 | 0.158633537 | 0.247336862 | -2.015450827 |
| Maob | 1 | 0.039235921 | | 4 | 0.158633537 | 0.247336862 | -2.015450827 |
| Ppp1r35 | 1 | 0.039235921 | | 4 | 0.158633537 | 0.247336862 | -2.015450827 |
| RGD1307182 | 1 | 0.039235921 | | 4 | 0.158633537 | 0.247336862 | -2.015450827 |
| 3-Sep | 1 | 0.039235921 | | 4 | 0.158633537 | 0.247336862 | -2.015450827 |
| Slc51b | 1 | 0.039235921 | | 4 | 0.158633537 | 0.247336862 | -2.015450827 |
| Sorcs1 | 1 | 0.039235921 | | 4 | 0.158633537 | 0.247336862 | -2.015450827 |
| Tmem114 | 1 | 0.039235921 | | 4 | 0.158633537 | 0.247336862 | -2.015450827 |
| Zbtb8b | 1 | 0.039235921 | | 4 | 0.158633537 | 0.247336862 | -2.015450827 |
