# Supplementary material for: Involvement of tumor necrosis factor alpha in steroid-associated osteonecrosis of the femoral head: friend or foe?
Source: Stem Cell Res Ther. 2019 Jan 3;10:5. doi: 10.1186/s13287-018-1112-x (PMC6318982; doi:10.1186/s13287-018-1112-x)
Supplement: Supplementary file 3 — Table S3. Specific primers for bisulfite sequencing PCR and ChIP-PCR. (DOCX 15 kb) [file 13287_2018_1112_MOESM3_ESM.docx]

**Table S3. Specific primers for bisulfite sequencing PCR and ChIP-PCR**

| Name | Sequence（5'-3'） |
| --- | --- |
| rRunx2-methy-F | GGGTGAAGAGTGTTTAGAAGTTAAT |
| rRunx2-methy-R | ATCCCTCCAACTCTTTACCAAATA |
| hRunx2-methy-F | AGAGTAAGGGGGAAAAGTTATAGTG |
| hRunx2-methy-R | AAAAACACTCACTAACTCTATTAATCTC |
| rRunx2- ChIP-F | CTGGCGATCACCTCCATCC |
| rRunx2- ChIP-R | GCATCACAACAGCCCACAAAG |

r, rat; h, human; F, forward; R, reverse
