# Supplementary figures and images for: Involvement of tumor necrosis factor alpha in steroid-associated osteonecrosis of the femoral head: friend or foe?
Source: Stem Cell Res Ther. 2019 Jan 3;10:5. doi: 10.1186/s13287-018-1112-x (PMC6318982; doi:10.1186/s13287-018-1112-x)

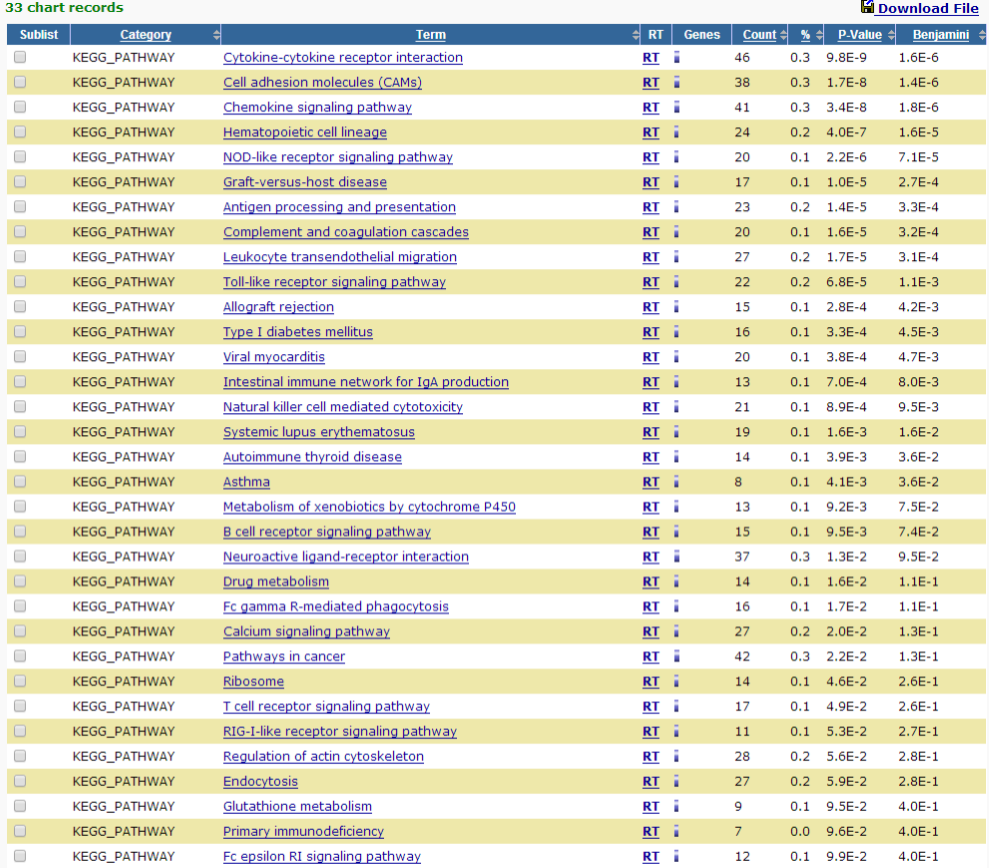

Supplement: Supplementary file 4 — Figure S1. The KEGG (Kyoto Encyclopedia of Genes and Genomes) analysis of enriched signaling pathways in TNFα-treated rMSCs. (TIF 562 kb) [file 13287_2018_1112_MOESM4_ESM.tif]
